# Triage Accuracy of Symptom Checker Apps: 5-Year Follow-up Evaluation

**DOI:** 10.2196/31810

**Published:** 2022-05-10

**Authors:** Malte L Schmieding, Marvin Kopka, Konrad Schmidt, Sven Schulz-Niethammer, Felix Balzer, Markus A Feufel

**Affiliations:** 1 Institute of Medical Informatics Charité - Universitätsmedizin Berlin Freie Universität Berlin and Humboldt-Universität zu Berlin Berlin Germany; 2 Cognitive Psychology and Ergonomics Department of Psychology and Ergonomics Technische Universität Berlin Berlin Germany; 3 Institute of General Practice and Family Medicine Jena University Hospital, Germany Jena Germany; 4 Institute of General Practice and Family Medicine Charité - Universitätsmedizin Berlin Freie Universität Berlin and Humboldt-Universität zu Berlin Berlin Germany; 5 Division of Ergonomics Department of Psychology and Ergonomics Technische Universität Berlin Berlin Germany

**Keywords:** digital health, triage, symptom checker, patient-centered care, eHealth apps, mobile phone

## Abstract

**Background:**

Symptom checkers are digital tools assisting laypersons in self-assessing the urgency and potential causes of their medical complaints. They are widely used but face concerns from both patients and health care professionals, especially regarding their accuracy. A 2015 landmark study substantiated these concerns using case vignettes to demonstrate that symptom checkers commonly err in their triage assessment.

**Objective:**

This study aims to revisit the landmark index study to investigate whether and how symptom checkers’ capabilities have evolved since 2015 and how they currently compare with laypersons’ stand-alone triage appraisal.

**Methods:**

In early 2020, we searched for smartphone and web-based applications providing triage advice. We evaluated these apps on the same 45 case vignettes as the index study. Using descriptive statistics, we compared our findings with those of the index study and with publicly available data on laypersons’ triage capability.

**Results:**

We retrieved 22 symptom checkers providing triage advice. The median triage accuracy in 2020 (55.8%, IQR 15.1%) was close to that in 2015 (59.1%, IQR 15.5%). The apps in 2020 were less risk averse (odds 1.11:1, the ratio of overtriage errors to undertriage errors) than those in 2015 (odds 2.82:1), missing >40% of emergencies. Few apps outperformed laypersons in either deciding whether emergency care was required or whether self-care was sufficient. No apps outperformed the laypersons on both decisions.

**Conclusions:**

Triage performance of symptom checkers has, on average, not improved over the course of 5 years. It decreased in 2 use cases (advice on when emergency care is required and when no health care is needed for the moment). However, triage capability varies widely within the sample of symptom checkers. Whether it is beneficial to seek advice from symptom checkers depends on the app chosen and on the specific question to be answered. Future research should develop resources (eg, case vignette repositories) to audit the capabilities of symptom checkers continuously and independently and provide guidance on when and to whom they should be recommended.

## Introduction

### Background

The use of patient-facing clinical decision support tools has become more and more prevalent in recent years. Tools assisting laypersons in their self-assessment on whether and where to seek professional medical care and for what diagnoses based on the users’ input of symptoms and medical history are termed symptom checkers. Especially at the beginning of the COVID-19 pandemic, such tools were developed to assist patients in deciding whether to call emergency services with symptoms indicative of a COVID-19 infection or whether self-isolation is required [1-4]. Although the 2021 World Health Organization global report on artificial intelligence for health [5] mentions symptom checkers explicitly only in this narrow context of outbreak response, symptom checkers have been available (and researched [6]) for more than a decade and typically address a broader spectrum of diseases.

Symptom checkers are becoming increasingly popular, with approximately 13% of the German adult population already having consulted an app for self-diagnosis [7]. Such apps are commonly used as a means of self-information and guidance through the health care system [8], although other potential use cases such as syndromic surveillance have been described as well [9,10]. Notably, some integrated delivery networks (health care networks) in the United States have begun to incorporate symptom checkers as a service for their members, be it for educational purposes or to improve their members’ experience of their patient journey, for example, by guidance on where and how urgently to seek care within the network on the symptom checker’s suggestion [11-13]. A recent study among Finnish primary care leaders of institutions integrating symptom checkers into their services demonstrates their support for the use of these systems [14]. Despite their popularity, symptom checkers also face concerns from both patients and health care professionals [15-17]. Insufficient accuracy of the advice provided is a commonly raised concern.

Although no clear framework has yet been established on evaluating the accuracy of symptom checker apps’ advice [18], a common first approach has been to test such systems on short fictitious patient descriptions (case vignettes), mirroring an approach to assess the reliability of diagnostic decision support systems for health care professionals [19,20], variability of initial diagnostic impressions [21], and diagnostic ability among physicians [22]. Independent studies using this approach suggest that advice from most symptom checker apps is rather unreliable, both for diagnosis and triage (ie, the assessment of urgency) [23-26]. A recent study with a slightly different approach, assessing 2 symptom checkers on information abstracted from medical records, came to the same conclusion [27]. Accordingly, 2 (systematic) reviews on the currently available evidence advise against using web-based triage systems in lieu of traditional urgency assessment means and emphasize the clinical risks that symptom checker use might pose [28,29].

### Objective

A key source of these and other reviews [30,31] on symptom checkers is a study by Semigran et al [23] published in 2015. They found that symptom checkers were rather risk averse at the time and reported an aggregated triage accuracy of 57% and diagnostic accuracy of providing the correct diagnostic suggestion first at 34%. A recent study assessing 4 symptom checkers on ophthalmologic case vignettes in 2018 and 2020 suggests that the capabilities of symptom checkers have not improved during this time frame [25]. Although other relevant studies competitively comparing symptom checkers’ accuracy have been published since 2015 [24,32,33], their chosen methodology (eg, sampling of apps and definition of triage levels) hinders a direct comparison with the data from the study by Semigran et al [23]. Thus, our study aims to revisit the landmark study by Semigran et al [23] to investigate whether and how symptom checkers’ capabilities have evolved since 2015 and how they compare with human decision makers.

## Methods

### Data Collection on Symptom Checker Performance in 2020

#### Search and Selection Criteria for Symptom Checkers

Between February and March 2020, we systematically searched for symptom checker apps and websites capable of providing triage advice following the approach of Semigran et al [23]. To identify smartphone apps, we entered *symptom checker* and *medical diagnoses* as keywords in Google Play Store and the US, UK, and German versions of the Apple App Store and screened the first 240 results provided using the same cutoff as Semigran et al [23]. Symptom checker apps had to be freely available in English. We excluded apps that did not provide triage advice or only addressed specific complaints (eg, skin conditions) or specialties (eg, apps for orthopedics). Unlike Semigran et al [23], we also dismissed apps when the number of downloads was <100,000 or when the app had received unsatisfactory reviews (<4 stars), as both features indicate that the app might not be used commonly.

To identify web-based symptom checkers, we searched Google and Google Scholar with the same keywords and screened the first 300 results. Symptom checkers that were included in the study by Semigran et al [23] were searched by their name and included if still publicly available. Symptom checkers mentioned in other scientific studies or known to the authors but not found during the search strategy were included if they met the inclusion criteria as described earlier.

#### Modification of Clinical Vignettes

To analyze the diagnostic and triage accuracy of symptom checker apps, we used the same 45 short descriptions of fictitious patients and their complaints (*case vignettes*) that were used in the original study by Semigran et al [23]. As the interpretation of a vignette may change if it does not include a particular piece of information that is requested by a symptom checker (eg, the chief complaint’s duration), we used several measures to augment the case description and, thus, improve the comparability of vignettes across the different symptom checkers. First, 2 authors (KS and MS), both physicians, complemented indication-specific details that they anticipated to be relevant for triage decisions by advanced symptom checkers, such as the onset of the chief complaint or the severity of pain. In addition, each case vignette was assigned a chief complaint as some symptom checkers require this as input. When applicable, we adopted the chief complaint assigned by Hill et al [24], a 2020 study that also built upon the vignette sample from the study by Semigran et al [23]. However, we retained the gold standard solutions for the correct diagnosis and the 3-tiered triage-level definitions as defined in the index study [23].

#### Assessing Diagnostic and Triage Accuracy of Symptom Checker Apps in 2020

A research assistant with no clinical training entered the case vignettes into the symptom checker apps between June and December 2020. In advance, a set of rules was defined on how to handle ambiguity during data entry and outcome interpretation; for example, when symptom checkers requested information that was not provided by the vignette or an app’s diagnostic suggestions were synonyms or umbrella terms for the gold standard diagnostic solution (Multimedia Appendix 1 [23,24,27,34-36]). Importantly, when a symptom checker app linked its triage advice to its diagnostic suggestions, we rated the triage advice for the first diagnosis it listed, assuming that this is the diagnosis that the app considers most likely and therefore the triage advice most relevant. This rule marks an exception from our general approach to retain the procedure of Semigran et al [23]. Semigran et al [23] used the most urgent triage level when the suggested diagnoses were linked with different triage suggestions. They argued that “in almost all of the cases the most urgent triage suggestion was listed first” [23]. However, we did not observe this and considered the triage advice linked to the diagnosis listed first. However, this divergence only affects 3 apps (*K Health*, *HealthTap*, and *WebMD*), as most apps provide triage advice not linked to diagnostic suggestions or also provide an overall triage appraisal.

As the apps use different classifications of triage urgency, we mapped all triage advice definitions of the assessed symptom checkers into the 3 categories that were defined by Semigran et al [23] (ie, emergency, nonemergency, and self-care). When symptom checkers provided triage advice that could not be matched to the 3 categories (eg, if a symptom checker identified emergency cases but could not specify whether self-care was sufficient or nonemergency care should be advised and when it deemed emergency care unnecessary), unspecified answers were counted as incorrect. To control for this decision, we conducted our main analyses twice, excluding and including such symptom checkers, and we report or provide both in the Multimedia Appendix 1.

### Comparator Data Sets for Symptom Checker Performance

We compared our data on symptom checker performance in 2020 with three publicly available data sets: (1) Semigran et al [23] (the index study) evaluated the diagnostic and triage accuracy of 23 symptom checker apps in 2015 using 45 case vignettes; (2) Hill et al [24,34] evaluated 36 symptom checker apps in 2020 on 48 clinical vignettes, using some of the vignettes compiled by Semigran et al [23] and new case vignettes; and (3) Schmieding et al [35] evaluated laypersons’ abilities to triage the same 45 case vignettes used by Semigran et al [23]. Although Semigran et al [23] used a 3-tiered classification of triage levels to set the gold standard solution (emergency care required, nonemergency care required, and self-care appropriate) and the study by Schmieding et al [36] retained this classification, the study by Hill et al [24] used a 4-tiered classification of triage levels (emergency, urgent, nonurgent, and self-care), thereby hindering a direct comparison of triage capability with the other data sets. Not all symptom checkers included in the first and second data sets and gave advice on both triage and diagnosis. In addition, in both studies, some symptom checkers never returned *self-care appropriate* as triage advice. A detailed description of these data sets can be found in the Multimedia Appendix 1. We made our data set publicly available via a web-based open data repository [37].

### Data Analysis

Data were cleaned and explored with R (version 4.0.0) [38] and the *tidyverse* packages [39]. Figures were created using the package *ggplot2* [40].

#### Direct Comparison of Symptom Checker Triage Performance Between 2015 and 2020

We defined the triage accuracy of a symptom checker app as the proportion of vignettes to which a symptom checker app provided the correct triage advice in relation to all vignettes to which the app provided triage advice. In other words, vignettes that a symptom checker could not triage were omitted from the denominator. Given that our data from 2020 was based on the same 45 case vignettes and we retained the same definition of urgency levels and the gold standard solutions as set by Semigran et al [23], a direct comparison between these data sets was possible to assess the evolution of symptom checkers’ triage capability between 2015 and 2020.

We calculated the median and IQRs of the apps’ triage accuracies for both data sets. To assess whether symptom checker apps were still as risk averse as reported in 2015, we calculated the odds of overtriage to undertriage, where overtriage refers to advice to seek a more urgent level of care than necessary, and undertriage refers to advice to seek care with less urgency than appropriate. In addition, we created confusion tables outlining which triage advice was provided during the evaluations of case vignettes from each of the 3 triage levels. In both the analysis of risk aversion and the confusion table, we excluded apps that did not provide self-care advice in our results reported here. For example, we excluded the symptom checker *iTriage* in this analysis, as it always advised to seek emergency care in the data from the study by Semigran et al [23] and thus potentially masks an interesting trend observable in those apps capable of providing triage advice for all 3 triage levels. Analyses including all apps can be found in the Multimedia Appendix 1.

#### Comparison of Triage Accuracy for Binary Triage Decisions

A 3-tiered triage classification as used by Semigran et al [23] (emergency, nonemergency, and self-care) and retained for our data collection comes with 2 downsides. First, a direct comparison of results from studies with different classifications of urgency levels is hindered, and second, common metrics of signal detection theory (eg, sensitivity and specificity) cannot be calculated. To facilitate a comparison of triage accuracy across studies with different triage definitions (eg, emergency, urgent care, nonurgent care, and self-care, as used in the study by Hill et al [24]), we created 2 binary triage accuracy measures: whether a symptom checker (or layperson) can differentiate between cases requiring emergency care (decision 1) and between cases where self-care was sufficient or professional medical care should be sought (decision 2). These 2 measures represent common triage decisions users of symptom checkers face [8]. Accordingly, case vignettes with gold standard urgency levels of *urgent care*, *nonemergency care*, *nonurgent care*, and *self-care* are counted as not requiring emergency care, whereas all urgency levels except *self-care* were counted as requiring professional health care. We calculated accuracy, sensitivity, and specificity for each measure and juxtaposed the median and IQR of apps based on the 4 data sets we compared.

#### Comparison of Diagnostic Accuracy

We assessed the evolution of diagnostic accuracy of symptom checkers by juxtaposing median and IQRs of 3 measures of diagnostic accuracy, abbreviated as M1, M10, and M20: they are defined as the proportions of case vignettes a symptom checker assessed where it suggested the gold standard diagnosis first (M1) within the first 10 (M10) or within the first 20 (M20) diagnostic suggestions it gave. We report M1 for all 3 data sets on symptom checkers, M10 for the Hill et al [24,34] and our data set, and the M20 measure for the Semigran et al [23] data set only.

#### Association Between Diagnostic and Triage Accuracy

Not all but many symptom checker apps (14/22, 64% in the data set sampled by us; 11/23, 48% in the data set sampled by Semigran et al [23]; and 8/36, 22% in the data set sampled by Hill et al [24,34]) provide both diagnostic and triage advice. As users approach symptom checker apps for different reasons—for example, some people aim at self-diagnosis, whereas others are looking for guidance through the health care system [8]—we wondered whether symptom checker apps either tend to perform well or poorly in both use cases or whether apps can provide helpful information on one of these questions but not the other. Thus, we explored whether the triage accuracy of these apps was linked to their diagnostic performance by analyzing the association of triage accuracy and diagnostic accuracy with linear models, 1 for each of the 3 samples of symptom checkers, and calculated the unadjusted *R*² value as a measure of variance explained. We further determined how commonly symptom checkers erred in their triage appraisal despite suggesting the correct diagnosis first. A high proportion indicates that symptom checkers grasp the case presentation but struggle with linking the correct triage level to the case presentation, for example, by providing overcautious triage advice despite having correctly identified a diagnosis of low urgency.

## Results

### Study Sample

Our systematic search returned 22 symptom checkers capable of providing triage advice, 14 (64%) of which also suggested diagnoses. Approximately 23% (5/22) of symptom checkers (*K Health*, *Isabel*, *Symcat*, *Everyday Health*, and *WebMD*) differentiated only 2 triage levels—emergency care and nonemergency care—whereas the other symptom checkers included *self-care* as potential triage advice. As most of the 22 symptom checkers were unable to evaluate every case vignette (eg, as their scope was limited to adult or pediatric patients), our assessment of the apps on 45 case vignettes yielded a total of 796 unique triage evaluations (22 apps, with a median of 40 evaluations per app and an IQR of 11) and 520 unique diagnostic evaluations (14 apps, with a median of 39 evaluations per app and an IQR of 15). Tables S1-S3 in Multimedia Appendix 1 list the retrieved symptom checker apps and denote their individual triage and diagnostic performance.

### Comparison of Triage Accuracy

#### Direct Comparison of Symptom Checkers’ Triage Accuracy in 2015 and 2020

**Figure 1:**
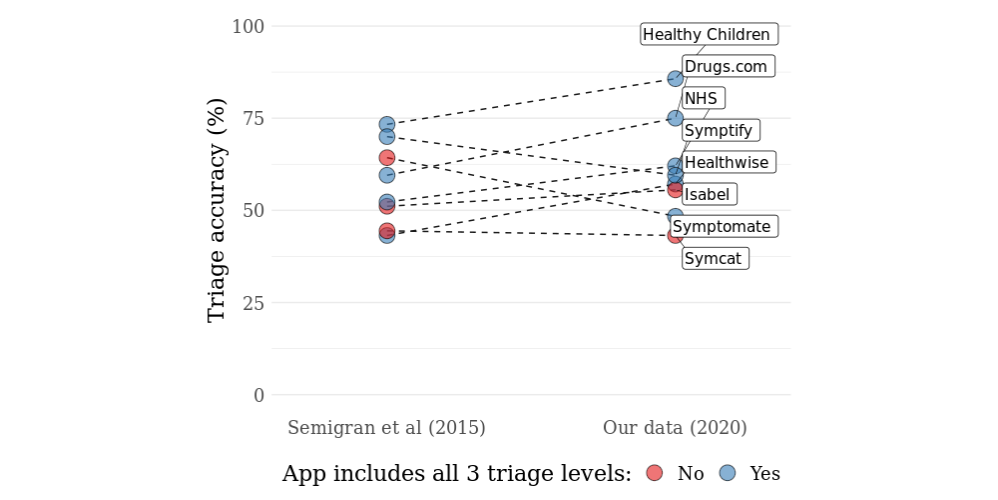
Overall triage accuracy of 8 symptom checkers included in both samples (2015 and 2020) and assessed on the same 45 case vignettes in 2015 and 2020. Data on symptom checker accuracy for 2015 are taken from a study by Semigran et al [23]. Of the 8 symptom checkers, 3 never recommended self-care as triage level (colored in red) in 2015 and 2 in 2020. One symptom checker (Symptomate) never recommended self-care in the 2015 study by Semigran et al [23] but provides such recommendations in 2020, as indicated both in our data and reported by Hill et al [24,35]. NHS: National Health Service.

The median overall triage accuracy of all symptom checkers in our data set from 2020 (55.8%, IQR 15.1%; N=22) is close to the median triage accuracy of the apps in 2015 by Semigran et al [23] (59.1%, IQR 15.5%; N=15). The medians remain similar when excluding apps that never advise seeking self-care (Table 1). Most of those apps included both in the sample of Semigran et al [23] and our sample (5/8, 63%) improved their overall triage accuracy on the set of 45 case vignettes (Figure 1).

**Table 1:** Overall triage accuracy of symptom checker apps in 2015 (data from a study by Semigran et al [23]; N=15) and 2020 (data collected by us; N=22).

Sample of symptom checker apps	Overall triage accuracy				
	2015			2020	
	Values (%), median (IQR)	Values, n (%)	Values (%), median (IQR)		Values, n (%)
All triaging apps included in the respective study	59.1 (51.7-67.1)	15 (100)	55.8 (47.8-62.9)		22 (100)
Subset of apps included in both studies	55.9 (49.4-65.7)	8 (53)	58.3 (53.8-65.3)		8 (36)
Subset of apps capable of providing self-care triage advice	59.5 (53.3-70.7)	11 (73)	59.5 (50.0-64.4)		17 (77)

#### Confusion Matrices for Triage Advice

The 2015 sample of 11 apps, providing triage advice and including all 3 urgency levels, more commonly erred by overtriaging a case vignette than by undertriaging (odds 2.82:1 and 110:39, respectively). The ratio of overtriage to undertriage was less for the respective sample of 17 apps in 2020 (odds 1.11:1 and 131:118, respectively). In comparison with the sample of 2015, the sample of 2020 less frequently mistook self-care cases and nonemergency cases for emergencies, whereas at the same time more often misclassified emergencies as nonemergencies (Tables 2 and 3).

Confusion matrices, including the case evaluations by those apps that did not provide triage advice on self-care, can be found in the Multimedia Appendix 1 (Tables S4 and S5). They show the same general trend as reported here.

**Table 2:** Confusion matrix of triage advice of 11 symptom checker apps assessed in 2015 by Semigran et al [23].

Triage recommendation provided by the symptom checker app	Gold standard solution of the triage level for the case vignette (15 case vignettes per category), n (%)		
	Emergency (n=130 evaluations)	Nonemergency (n=128 evaluations)	Self-care (n=127 evaluations)
Emergency care	103 (79.2)	41 (32)	23 (18.1)
Nonemergency	22 (16.9)	74 (57.8)	46 (36.2)
Self-care	5 (3.8)	13 (10.1)	58 (45.6)

**Table 3:** Confusion matrix of triage advice of 17 symptom checker apps assessed in 2020 on the same 45 case vignettes as used by Semigran et al [23] in 2015.

Triage recommendation provided by the symptom checker app	Gold standard solution of the triage level for the case vignette (15 case vignettes per category), n (%)		
	Emergency (n=202 evaluations)	Nonemergency (n=205 evaluations)	Self-care (n=193 evaluations)
Emergency care	116 (57.4)	26 (12.6)	6 (3.1)
Nonemergency	80 (39.6)	147 (71.7)	99 (51.2)
Self-care	6 (2.9)	32 (15.6)	88 (45.5)

#### Symptom Checkers’ Triage Capability on Binary Triage Decisions in 2015 and 2020

**Figure 2:**
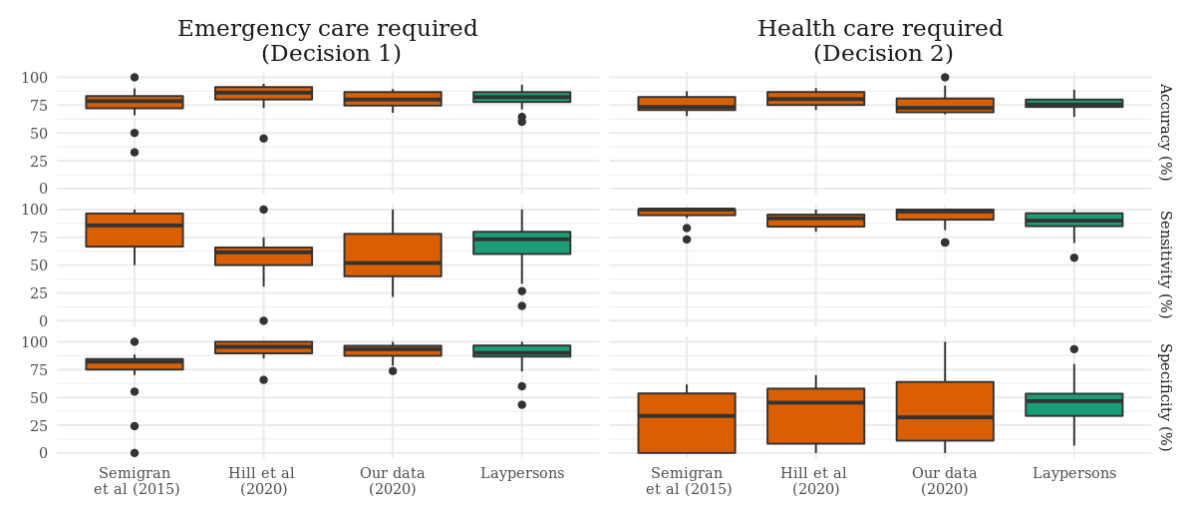
Accuracy, sensitivity, and specificity of symptom checker apps and laypersons for 2 binary triage decisions on whether emergency care is required and whether professional medical care is required at all. Data for symptom checkers are taken from Semigran et al [23], Hill et al [24,35], and our own data collection. Data on laypersons are taken from Schmieding et al [36].

The median accuracy of the apps in deciding whether emergency care is necessary (decision 1) in 2015 (78.6%, IQR 72.1%-83.1%) was similar to our re-evaluation in 2020 (80%, IQR 74.6%-86.8%; Figure 2). The same holds true regarding the median accuracy for decision 2, whether medical care should be sought or self-care is sufficient (73.3%, IQR 70.5%-82.3% vs 72.6%, IQR 68.5%-81%). Differences between the apps’ triage performance in 2015 and 2020 appear when comparing the sensitivity and specificity for detecting emergencies. In 2015, the median app correctly spotted 85.7% (IQR 66.7%-96.4%) of the emergencies (sensitivity), with a median specificity of 82.1% (IQR 75%-84.5%). In comparison, in our 2020 data, the median app spotted 51.9% (IQR 40%-78.2%) of the emergencies (sensitivity) and attained a specificity of 93.3% (IQR 87.4%-96.4%). Comparing the data from studies by Semigran et al [23] and Hill et al [24,34] reveals the same trend, with a low sensitivity to identify emergencies (61.5%, IQR 50%-65.9%) and high specificity to rule them out (95.5%, IQR 89.6%-100%) in 2020. Such a trend cannot be detected regarding decision 2, whether professional medical care (health care) is required, as sensitivity and specificity scores from 2015 are close to those from 2020 (Figure 2).

#### Symptom Checkers’ Triage Capability on Binary Triage Decisions in 2020 Compared With Laypersons

The sample of 22 symptom checkers assessed in 2020 performed very similarly to laypersons’ triage accuracy (Figure 2) [36]. However, few apps managed to outperform laypersons on binary triage decisions. Concerning decision 1, whether emergency health care should be sought or not, 18% (4/22) of apps (*Mayo*, *Ada*, *Isabel*, and *Healthwise*) showed a higher accuracy, sensitivity, and specificity than the median layperson (accuracy 82.2%, sensitivity 73.3%, and specificity 90%). Concerning decision 2, whether professional medical care should be sought, 23% (5/22) of apps (*Healthy Children*, *NHS*, *Drugs.com*, *Healthily*, and *Earlydoc*) managed to outperform the median layperson’s accuracy (75.9%), sensitivity (90%), and specificity (46.7%; Table S2 in Multimedia Appendix 1).

### Comparison of Diagnostic Accuracy

**Figure 3:**
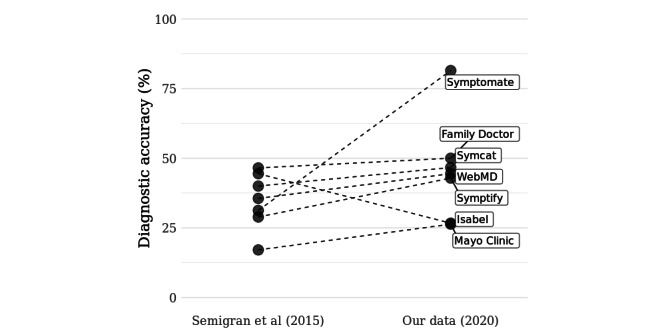
Overall diagnostic accuracy (correct diagnosis listed first, M1) of 7 symptom checkers included in both samples (2015 and 2020) and assessed on the same 45 case vignettes in 2015 and 2020. Data on symptom checker accuracy for 2015 are taken from Semigran et al [23].

The 64% (14/22) of symptom checkers that provided diagnostic advice in 2020, on average, provided the correct diagnostic suggestion first (M1) for approximately half the case vignettes assessed, and two-thirds of the time, the correct diagnosis was listed among the first 10 suggestions on average (M10; Table 4). The M1 score is higher than the sample median diagnostic accuracies reported by Semigran et al [23] in 2015. In line with this, of the 7 symptom checker apps providing diagnostic suggestions and included in the samples by both Semigran et al [23] and our study, the majority (6/7) improved their M1 diagnostic accuracy (Figure 3). Hill et al [24,34] reported median diagnostic accuracy scores in 2020 closer to those of Semigran et al [23] for M1 diagnostic accuracy.

**Table 4:** Diagnostic accuracy of symptom checkers as reported by Semigran et al [23] in 2015, Hill et al [24,34], and our data set from 2020^a^.

Metric of diagnostic accuracy	Diagnostic accuracy of symptom checkers (%), median (IQR)		
	Semigran et al [23] (n=19 apps)	Hill et al [24,34] (n=24 apps)	Our data (n=14 apps)
M1	35.5 (30.0-40.0)	34.3 (26.5-40.1)	45.5 (37.5-51.7)
M10	—^b^	59.2 (40.5-70.8)	71.1 (60.9-76.9)
M20	55.8 (45.2-73.6)	—	—

^a^Diagnostic accuracy as reported by Hill et al [24,34] is based on a different but overlapping set of case vignettes. M1, M10, and M20 abbreviate the proportion of case vignettes a symptom checker assessed where it suggested the gold standard diagnosis first (M1) within the first 10 (M10) or within the first 20 diagnostic suggestions (M20). The table displays the median and IQR values on these 3 metrics of the 3 samples of symptom checkers.

^b^Not available: Semigran et al [23] presented values only for M1, M3, and M20. Hill et al [24,34] and our data collection disregarded diagnostic suggestions beyond the first 10 suggestions.

### Relation Between Diagnostic and Triage Accuracy

**Figure 4:**
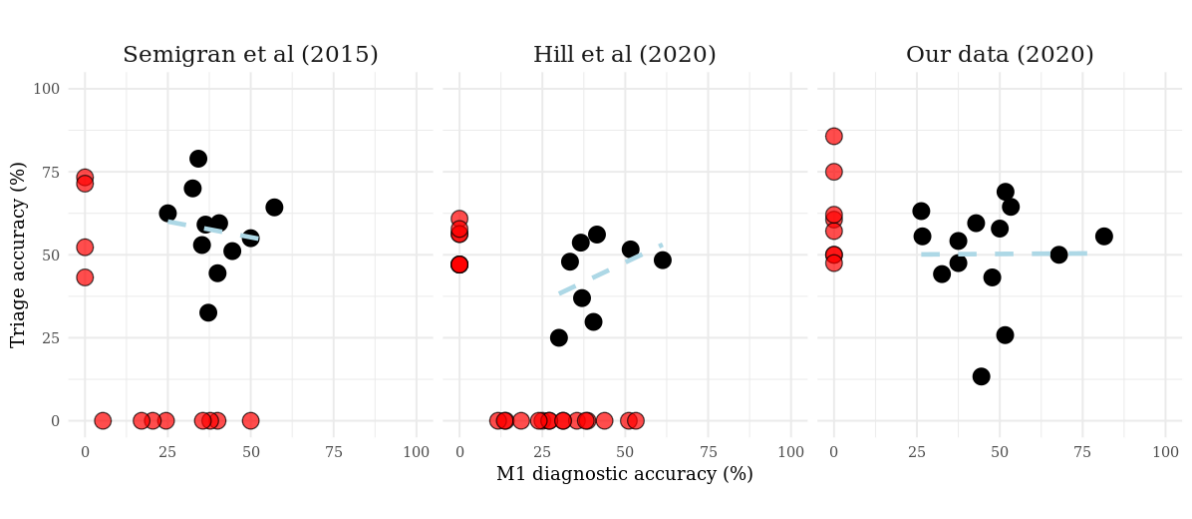
Association between M1 diagnostic accuracy (proportion of case vignettes to which the app provided the correct diagnosis first, as percentage) and triage accuracy. Every dot represents a symptom checker app. Red dots represent apps that provide either only triage or only diagnostic advice. Data for symptom checkers are taken from studies by Semigran et al [23], Hill et al [24,35], and our own data collection.

Considering those apps that provided both diagnostic and triage advice, the proportion of wrong triage assessments when the correct diagnosis was suggested first is 37.7% (57/151) for the study by Semigran et al [23], 37.6% (88/234) in our data, and 46.4% (58/125) in the data provided by Hill et al [24,34]. Accordingly, the individual symptom checker app’s top 1 diagnostic and triage accuracy does not correlate well with low unadjusted *R*² values (0.018, 0.175, and <0.001 for the Semigran et al [23], Hill et al [24,34], and our data sets, respectively; Figure 4). In the study by Semigran et al [23], most of these erroneous triage assignments were overtriage errors (52/57, 91%), whereas this proportion was lower in our data (46/88, 52%) and in that of the Hill et al [24,34] study (34/58, 58%). Concerning those evaluations where symptom checkers got the diagnosis right but allocated the wrong triage levels, many of the errors were because of a false appraisal of whether emergency care was necessary or not (Semigran et al [23] study: 29/57, 51%; Hill et al [24,34] study: 18/58, 31%; and our data: 24/88, 27%).

## Discussion

### Principal Findings

#### Evolution of Triage Capability of Symptom Checker Apps

Our study assesses how the triage and diagnostic capability of symptom checkers evolved from 2015 to 2020. A direct comparison between the data from Semigran et al [23] and the data collected by us in 2020 indicates that overall triage accuracy has not improved over the course of 5 years with respect to the same set of case vignettes. This holds true even when we look beyond the overall triage accuracy rate with 3 triage levels (emergency, nonemergency, and self-care) and instead assess the apps’ accuracy to advise on seeking emergency versus no emergency care (decision 1) or seeking medical care versus not care at all (decision 2).

However, the pattern where symptom checker apps perform well and poorly seems to have changed. In 2015, apps were more risk averse (ie, they detected emergencies reliably and tended to overtriage less urgent cases), whereas both our data and the data from Hill et al [24,34] show that in 2020, apps tended to be less risk averse and missed more emergencies. The ability to reliably detect emergencies (the sensitivity to identify emergencies) can be considered the most important metric for assessing a symptom checker’s safety. On the other hand, the ability to correctly spot those cases where self-care is sufficient (ie, the specificity to correctly rule out cases when professional health care is not necessary) can be considered the most important metric for assessing the usefulness of an app to both its users and the health care system, as this is the most difficult decision for laypersons [36], providing a great potential to disburden health care services. According to the data of both Hill et al [24,34] and our own study, symptom checkers still did not perform particularly well on both metrics in 2020. Comparing the distribution of triage errors (Table 3) with that of medical laypersons, as reported by Schmieding et al [36], we observe that the triage behavior of symptom checkers and medical laypersons have aligned. Thus, we consider it a pressing question whether symptom checkers can enhance laypersons’ decision-making when both their accuracy and direction of errors are similar. The importance of this question is supported by an experimental study demonstrating that most laypersons do not change their triage assessment after an internet search, and when they do, this change is as commonly correct as it is incorrect [41].

However, it must be noted that among our sample of apps, some defy the general trend and show high rates of accuracy, sensitivity, and specificity for either but not both of the binary triage decisions, indicating that they are potentially beneficial to their users when approached with the appropriate question (Table S2, Multimedia Appendix 1).

#### Evolution of Diagnostic Capability of Symptom Checker Apps

The rate of correct diagnoses being presented first (M1 diagnostic accuracy) by symptom checkers is still low, with a median of <50%. Only 2 symptom checkers (*Everyday Health* and *Symptomate*) in our sample achieved an M1 diagnostic value near the M1 diagnostic accuracy of physicians on these case vignettes, reported at 72.1% by Semigran et al [42] in a study from 2016 (Table S3, Multimedia Appendix 1). However, it must be noted that both apps did not evaluate all 45 case vignettes, and thus, their observed diagnostic accuracy might be skewed by selection bias. However, in contrast to the evolution of triage capability, data from a study by Hill et al [24,34] and our own data hint at a slight improvement of diagnostic accuracy, as the correct diagnosis is now more commonly included in the top 10 suggestions (M10) than in the first 20 suggestions in 2015. However, it must be considered that these case vignettes have been publicly available, and therefore, app developers potentially trained their apps’ algorithms on these cases. Hence, the diagnostic accuracy for previously unpublished case vignettes might have increased differently or not all. In addition, as users are presumably most affected by the first or first few diagnostic suggestions, we question whether the observed increase in diagnostic capability already translates into an additional benefit noticeable to users.

#### Association Between Symptom Checker Apps’ Diagnostic and Triage Capability

All 3 data sets assessing symptom checker performance considered in this study indicate that correct diagnostic evaluation does not reliably translate into correct triage evaluation; that is, apps capable of diagnosing correctly are not necessarily good at triaging. Given that a large proportion of triage advice was incorrect even when the app correctly diagnosed the case (between 37% and 46%), a cause for the divergence of triage and diagnostic might be that app developers assign a wrong triage level to their diagnoses. However, as symptom checkers are developed by companies and agencies from different countries with potentially very different health care systems, the assignment of a specific triage level to a diagnosis might be wrong in one health care setting but correct in another. We observed that a considerable proportion of correctly diagnosed but incorrectly triaged case vignettes were incorrectly classified as (not) constituting an emergency. As we deem the variation between health care systems concerning the definition of what constitutes an emergency low, triage advice tailored to a specific health care system cannot be the only explanation for the observed discrepancies between triage and diagnostic capability.

### Limitations

The assessment of symptom checker performance in our study is based on clinical vignettes and thus comes with important limitations, which our study shares with other case vignette-based approaches to assess symptom checkers: patient users might enter their complaints less reliably and more ambiguously into apps compared with the mock users who enter information from clinical vignettes in a more structured fashion. A study by Jungmann et al [43] shows low interrater reliability among laypersons in entering information into symptom checker apps. Thus, vignette-based studies potentially have a poor ecological validity (ie, transferability to the real-world setting) and might overestimate the accuracy of symptom checkers when used by their target users—laypersons—in a real-world setting as they do not account for users’ variable capability to enter their symptoms without making errors.

Furthermore, researchers assign a correct solution (gold standard diagnosis and triage level) to each case vignette. However, there might be >1. These case vignettes commonly represent the first presentation of new complaints of a fictitious patient, and thus—similar to the real clinical setting—to definitely determine the correct diagnosis and most appropriate triage level, additional information might be required, such as imaging or laboratory findings. Thus, at the time of initial presentation, which is also the time when symptom checkers tend to be used, multiple options might be considered correct when more predictive information is not yet available.

In addition, even when symptom checkers achieve high accuracy, their true value to the users can only be fully estimated when taking into account the users’ own appraisal, prior knowledge, and trust in the symptom checker [41,44,45]. Thus, an evaluation of symptom checkers with case vignettes alone is a useful but only a first step to identify the best symptom checkers; in a second step, the best-in-class apps should then be further evaluated with study designs where patients enter their own complaints [46-48], and patient-reported outcomes and experience measures should be brought into focus.

Despite the limitations of vignette-based audit studies, we are convinced they will remain essential, as they provide a means for quick and potentially automatable evaluations of symptom checkers. With symptom checker software being updated on a regular basis and new apps continuously becoming available, we consider the vignette-based approach a necessary complement to more informative but also more costly and lengthy clinical studies. Thus, we advocate for the further development of the key resources of such studies—the clinical vignettes. We suggest the creation of a repository of vignettes not only based on real patient histories but also refined by a test–theoretical perspective and annotated by machine-readable codes (such as Systematized Nomenclature of Medicine–Clinical Terms semantic tags) to pave the way for automating the evaluation of those symptom checkers providing a suitable application programming interface.

Apart from the limitations deriving from the use of vignettes, our study comes with an additional set of specific limitations. As symptom checkers appear and disappear, the sample of retrievable symptom checkers varies over time. Of the 15 triaging symptom checkers assessed in 2015, only 8 (53%) were retrievable in 2020. In addition, although an implicit consensus on defining symptom checkers by their function emerges, it is difficult to definitely determine whether a *tool* can be considered a general-purpose symptom checker app and thus be included in studies. For example, the tool *Healthy Children* was considered a symptom checker app by Semigran et al [23] and Hill et al [24], although it merely presents a list of advice and descriptions of common diagnoses associated with single chief complaints without performing an input-based assessment of a patient’s complaints as most other tools do. For the purpose of comparability, we included *Healthy Children* and similar tools in our study as well, although we are aware that other researchers disregard these tools in their studies [32], arguably as such tools have not much in common with smartphone apps or web-based applications more reactive to user input and built on computational rather a tree-based algorithms, except their shared use case. Consequently, any attempt to assess the presumed population of symptom checker apps faces the difficulty that what constitutes a symptom checker is ill defined. To avoid rarely used, potentially poorly performing apps distorting the results, we excluded apps with few downloads or below a certain threshold of user rating scores. By doing so, the inclusion criteria for our study were more strict than in the study by Semigran et al [23]; however, the limitations certainly remain.

The heterogeneous definitions of triage levels potentially pose an additional, important limitation for all symptom checker comparison studies. Some studies on symptom checkers use only 2 triage levels [25], whereas others use as many as 6 [33]. By defining 2 binary metrics of triage accuracy rather than just 1, we could mitigate this limitation but only partially, as triage-level definitions can also be incongruent between studies as, for instance, the definition of *urgent care* by Hill et al [24] mostly covers what Semigran et al [23] define as *nonemergency* care but partially overlaps with the definition of *emergency care* by Semigran et al [23] as well. Thus, we recommend that future studies that aim to compare the triage capability of symptom checkers competitively also include binary triage measures that resemble real-life decisions (eg, should I call an ambulance or not and is self-care sufficient and safe?), in addition to more compartmentalized classifications of triage levels that can be tailored for the local health care system at best and arbitrary at worst. Although more compartmentalized classifications make a meaningful comparison between symptom checkers with different triage-level definitions more difficult, this approach does acknowledge that the potential real-world benefit of symptom checkers also lies in their ability to guide their users through the health care system with advice that is as specific as possible.

Finally, we consider our greatest limitation that a single mock user, a nonnative English speaker, compiled our data for symptom checker performance in 2020. We tried to minimize but certainly did not eliminate the risk that our comparative analyses are influenced by this by (1) complementing the case vignettes in advance with additional information we anticipated symptom checkers would prompt the mock user to provide and (2) defining rules on how to handle ambiguities in symptom checkers’ questions or in the vignettes.

### Conclusions

Assessing the capabilities of symptom checkers in a transparent and reproducible manner is challenging but necessary to gather independent and non–industry-funded evidence on these increasingly popular decision support tools for patients and laypersons. Our study compares 3 data sets on symptom checkers’ diagnostic and triage performance, 1 with data from 2015 and 2 with data from 2020. Taken together, they suggest that symptom checkers’ triage performance has, on average, not improved over the course of 5 years, and it potentially even decreased in the most important use cases (safe advice on when emergency care is required and when no health care is needed for the moment). Few highly performing apps managed to provide more reliable triage advice than an average layperson in one of those important 2 use cases. However, no symptom checker outperformed the laypersons in both use cases, and in general, symptom checkers’ triage behavior has become more similar to that of laypersons. Although some apps are good at both triaging and diagnosis, no general association between an app’s triage and diagnostic ability exists to date. In addition, the accuracy of advice does not only vary considerably between symptom checkers but also within a given symptom checker, as it may prove more reliable in appraising some categories of diseases than others [48]. Taken together, these findings highlight that the current value of symptom checkers heavily depends not only on the app system but also on the question (use case) with which it is approached, for instance, whether to seek care or, if so, where or for what. Thus, medical laypersons seeking useful decision support from symptom checkers face the complexity of choosing which tool to use for what. To aid the public in taking advantage of this emerging technology, future research should develop resources (eg, repositories of case vignettes) and frameworks with which symptom checkers’ performance can be evaluated continuously and independently. Together with research findings on how users integrate symptom checker’s advice into their decision-making, findings on these decision aid’s capabilities can provide valuable guidance as to when and to whom their use can be recommended.

## Appendix

Multimedia Appendix 1 Supplementary tables and details on methods.

## References

[1] Lunn PD, Timmons S, Julienne H, Belton CA, Barjaková M, Lavin C, McGowan FP (2021). Using decision aids to support self-isolation during the COVID-19 pandemic. Psychol Health.

[2] Zhang MW, Chow A, Ho RC, Smith HE (2021). An overview of commercially available apps in the initial months of the COVID-19 pandemic. Front Psychiatry.

[3] Judson TJ, Odisho AY, Neinstein AB, Chao J, Williams A, Miller C, Moriarty T, Gleason N, Intinarelli G, Gonzales R (2020). Rapid design and implementation of an integrated patient self-triage and self-scheduling tool for COVID-19. J Am Med Inform Assoc.

[4] Alanzi T (2021). A review of mobile applications available in the App and Google play stores used during the COVID-19 outbreak. J Multidiscip Healthc.

[5] World Health Organization (2021). Applications of artificial intelligence for health. Ethics and governance of artificial intelligence for health: WHO guidance.

[6] Farmer SE, Bernardotto M, Singh V (2011). How good is Internet self-diagnosis of ENT symptoms using Boots WebMD symptom checker?. Clin Otolaryngol.

[7] (2020). EPatient survey 2020. Health & Care Management.

[8] Meyer AN, Giardina TD, Spitzmueller C, Shahid U, Scott TM, Singh H (2020). Patient perspectives on the usefulness of an artificial intelligence-assisted symptom checker: cross-sectional survey study. J Med Internet Res.

[9] Elliot AJ, Kara EO, Loveridge P, Bawa Z, Morbey RA, Moth M, Large S, Smith GE (2015). Internet-based remote health self-checker symptom data as an adjuvant to a national syndromic surveillance system. Epidemiol Infect.

[10] Mehl A, Bergey F, Cawley C, Gilsdorf A (2020). Syndromic surveillance insights from a symptom assessment app before and during COVID-19 measures in Germany and the United Kingdom: results from repeated cross-sectional analyses. JMIR Mhealth Uhealth.

[11] Medical symptom checker. Sutter Health.

[12] (2019). Sutter health teams up with ADA health to improve patient care by delivering on-demand healthcare guidance. Sutter Health.

[13] Check your symptoms: who has the problem?. Kaiser Permanente.

[14] Laukka E, Kujala S, Gluschkoff K, Kanste O, Hörhammer I, Heponiemi T (2021). Leaders' support for using online symptom checkers in Finnish primary care: survey study. Health Informatics J.

[15] Aboueid S, Meyer S, Wallace JR, Mahajan S, Chaurasia A (2021). Young adults' perspectives on the use of symptom checkers for self-triage and self-diagnosis: qualitative study. JMIR Public Health Surveill.

[16] Kujala S, Hörhammer I, Hänninen-Ervasti R, Heponiemi T (2020). Health professionals' experiences of the benefits and challenges of online symptom checkers. Stud Health Technol Inform.

[17] (2019). Using technology to ease the burden on primary care. Healthwatch Enfield.

[18] Fraser H, Coiera E, Wong D (2018). Safety of patient-facing digital symptom checkers. Lancet.

[19] Berner ES, Webster GD, Shugerman AA, Jackson JR, Algina J, Baker AL, Ball EV, Cobbs CG, Dennis VW, Frenkel EP (1994). Performance of four computer-based diagnostic systems. N Engl J Med.

[20] Bond WF, Schwartz LM, Weaver KR, Levick D, Giuliano M, Graber ML (2012). Differential diagnosis generators: an evaluation of currently available computer programs. J Gen Intern Med.

[21] Yager J, Linn LS, Leake B, Gastaldo G, Palkowski C (1986). Initial clinical judgments by internists, family physicians, and psychiatrists in response to patient vignettes: I. Assessment of problems and diagnostic possibilities. Gen Hosp Psychiatry.

[22] Peabody JW, Luck J, Glassman P, Dresselhaus TR, Lee M (2000). Comparison of vignettes, standardized patients, and chart abstraction: a prospective validation study of 3 methods for measuring quality. JAMA.

[23] Semigran HL, Linder JA, Gidengil C, Mehrotra A (2015). Evaluation of symptom checkers for self diagnosis and triage: audit study. BMJ.

[24] Hill MG, Sim M, Mills B (2020). The quality of diagnosis and triage advice provided by free online symptom checkers and apps in Australia. Med J Aust.

[25] Ćirković A (2020). Evaluation of four artificial intelligence-assisted self-diagnosis apps on three diagnoses: two-year follow-up study. J Med Internet Res.

[26] Berry AC, Cash BD, Mulekar MS, Wang B, Melvin A, Berry BB (2017). Symptom checkers vs. doctors, the ultimate test: a prospective study of patients presenting with abdominal pain. Gastroenterology.

[27] Yu SW, Ma A, Tsang VH, Chung LS, Leung SC, Leung LP (2019). Triage accuracy of online symptom checkers for Accident and Emergency Department patients. Hong Kong J Emerg Med.

[28] Gottliebsen K, Petersson G (2020). Limited evidence of benefits of patient operated intelligent primary care triage tools: findings of a literature review. BMJ Health Care Inform.

[29] Wallace W, Chan C, Chidambaram S, Hanna L, Iqbal FM, Acharya A, Normahani P, Ashrafian H, Markar S, Sounderajah V, Darzi A (2021). The diagnostic and triage accuracy of digital and online symptom checker tools: a systematic review. medRxiv (forthcoming).

[30] Mueller J, Jay C, Harper S, Davies A, Vega J, Todd C (2017). Web use for symptom appraisal of physical health conditions: a systematic review. J Med Internet Res.

[31] Chambers D, Cantrell AJ, Johnson M, Preston L, Baxter SK, Booth A, Turner J (2019). Digital and online symptom checkers and health assessment/triage services for urgent health problems: systematic review. BMJ Open.

[32] Ceney A, Tolond S, Glowinski A, Marks B, Swift S, Palser T (2021). Accuracy of online symptom checkers and the potential impact on service utilisation. PLoS One.

[33] Gilbert S, Mehl A, Baluch A, Cawley C, Challiner J, Fraser H, Millen E, Montazeri M, Multmeier J, Pick F, Richter C, Türk E, Upadhyay S, Virani V, Vona N, Wicks P, Novorol C (2020). How accurate are digital symptom assessment apps for suggesting conditions and urgency advice? A clinical vignettes comparison to GPs. BMJ Open.

[34] Hill MG (2020). Appraisal of free online symptom checkers and applications for self-diagnosis and triage: an Australian evaluation. Edith Cowan University.

[35] Schmieding ML, Mörgeli R, Schmieding MA, Feufel MA, Balzer F (2021). Data set supplementing "Benchmarking triage capability of symptom checkers against that of medical laypersons: survey study". Zenodo.

[36] Schmieding ML, Mörgeli R, Schmieding MA, Feufel MA, Balzer F (2021). Benchmarking triage capability of symptom checkers against that of medical laypersons: survey study. J Med Internet Res.

[37] Schmieding ML, Kopka M, Schmidt K, Schulz-Niethammer S, Blazer F, Feufel M (2022). Data set on accuracy of symptom checker apps in 2020. Zenodo.

[38] R Core Team (2021). R: a language and environment for statistical computing. R Foundation for Statistical Computing.

[39] Wickham H, Averick M, Bryan J, Chang W, McGowan LD, François R, Grolemund G, Hayes A, Henry L, Hester J, Kuhn M, Pedersen T, Miller E, Bache S, Müller K, Ooms J, Robinson D, Seidel DP, Spinu V, Takahashi K, Vaughan D, Wilke C, Woo K, Yutani H (2019). Welcome to the Tidyverse. J Open Source Softw.

[40] Wickham H (2016). ggplot2: Elegant Graphics for Data Analysis.

[41] Levine DM, Mehrotra A (2021). Assessment of diagnosis and triage in validated case vignettes among nonphysicians before and after internet search. JAMA Netw Open.

[42] Semigran HL, Levine DM, Nundy S, Mehrotra A (2016). Comparison of physician and computer diagnostic accuracy. JAMA Intern Med.

[43] Jungmann SM, Klan T, Kuhn S, Jungmann F (2019). Accuracy of a chatbot (Ada) in the diagnosis of mental disorders: comparative case study with lay and expert users. JMIR Form Res.

[44] Woodcock C, Mittelstadt B, Busbridge D, Blank G (2021). The impact of explanations on layperson trust in artificial intelligence-driven symptom checker apps: experimental study. J Med Internet Res.

[45] Winn AN, Somai M, Fergestrom N, Crotty BH (2019). Association of use of online symptom checkers with patients' plans for seeking care. JAMA Netw Open.

[46] Martin SS, Quaye E, Schultz S, Fashanu OE, Wang J, Saheed MO, Ramaswami P, de Freitas H, Ribeiro-Neto B, Parakh K (2019). A randomized controlled trial of online symptom searching to inform patient generated differential diagnoses. NPJ Digit Med.

[47] Knitza J, Mohn J, Bergmann C, Kampylafka E, Hagen M, Bohr D, Morf H, Araujo E, Englbrecht M, Simon D, Kleyer A, Meinderink T, Vorbrüggen W, von der Decken CB, Kleinert S, Ramming A, Distler JH, Vuillerme N, Fricker A, Bartz-Bazzanella P, Schett G, Hueber AJ, Welcker M (2021). Accuracy, patient-perceived usability, and acceptance of two symptom checkers (Ada and Rheport) in rheumatology: interim results from a randomized controlled crossover trial. Arthritis Res Ther.

[48] Hennemann S, Kuhn S, Witthöft M, Jungmann SM (2022). Diagnostic performance of an app-based symptom checker in mental disorders: comparative study in psychotherapy outpatients. JMIR Ment Health.

