# Supplementary material for: Triage Accuracy of Symptom Checker Apps: 5-Year Follow-up Evaluation
Source: J Med Internet Res. 2022 May 10;24(5):e31810. doi: 10.2196/31810 (PMC9131144; doi:10.2196/31810)
Supplement: Multimedia Appendix 1 [file jmir_v24i5e31810_app1.docx]

Multimedia Appendix

# Description of comparator data sets

## Data Set from Semigran et al 2015

To approach the question whether symptom checker accuracy changed over time, we compared our data collected in 2020 to the previous study by Semigran et al, which reported the diagnostic and triage accuracy of symptom checker apps in 2015 [23].

Semigran et al [23] evaluated 23 symptom checker apps in total, 15 capable of providing triage advice and 19 providing diagnostic suggestions. Given that most of these triage apps (13/15) were not able to assess all 45 case vignettes (eg, some apps evaluated only pediatric cases and some only adult cases), these apps yielded a total of 532 triage evaluations. Four of 15 triage apps (*Symcat*, *Symptomate*, *Isabel*, *iTriage*) never recommended the least urgent triage level (self-care). Of the 15 triage apps, 8 were still available in 2020 and were included in our data set of 22 symptom checker apps. The 7 remaining triage apps are no longer publicly available, as has been reported before [27]. Because we used the same case vignettes as Semigran et al [23] and retained their definition of triage levels, we can directly compare the overall triage accuracy reported by Semigran et al [23] with the results of our analysis.

The 19 diagnosis apps Semigran et al evaluated yielded 770 diagnostic evaluations [23]. Semigran et al coded diagnostic accuracy with three metrics in their appendix [23]: whether the correct diagnosis (or a synonym) was listed first (M1), among the first ten (M10), or among the first 20 diagnostic suggestions (M20) [23]. To be able to compare the results, we used the same metrics for our analysis, but omitted the M20 metric, as only very few apps suggested that many diagnoses.

## Data Set from Hill et al 2020

Since Semigran et al published their case vignettes in 2015 [23], app developers were able to use them to train the decision algorithms of their apps and, by doing so, their apps could potentially achieve a higher performance on these specific case vignettes than on hitherto unknown ones. We sought to validate our findings on the evolution of symptom checker performance between 2015 and 2020 by comparing them with a more recent evaluation study, which included case vignettes that were published for the first time in 2020 [24,34].

In this study, Hill et al evaluated 36 symptom checkers, 16 of which provided triage advice and 24 provided diagnostic advice, yielding 688 triage evaluations and 1170 diagnostic evaluations [24,34]. Among their sample, 4 of the 16 triaging apps never suggested self-care for any vignette (*Isabel Healthcare*, *Everyday Health*, *Symcat*, *Doctor Diagnose*). Hill et al reported diagnostic accuracy with two metrics: whether the correct diagnosis (or a synonym) was listed first (M1) or among the first ten suggestions (M10) [24,34].

Unlike Semigran et al [23], Hill et al [24,34] used a four-tiered classification of triage levels (emergency, urgent, non-urgent and self-care). Thus, a direct comparison of triage accuracies with those reported by Semigran et al [23] and in our data was not possible. To be able to compare triage accuracies nonetheless, we defined two metrics for triage capability which can be calculated for all data sets and thereby allows for a comparison of triage capability results across these data sets, see Data Analysis below for details.

Finally, Hill et al [24,34] counted symptom checkers that were available through three platforms (eg, as web-based applications smartphone app in the Google App Store and *and* as app in the Apple Store) as three different symptom checkers. To reduce complexity, we counted such apps as one app for the purpose of our study if they performed the same across different platforms.

## Data Set from Schmieding et al 2021

A study from 2021 benchmarked the triage accuracy of symptom checker apps based on Semigran et al (2015) against that of medical laypersons [35]. The sample consisted of 91 US residents without professional medical background. As every participant assessed all 45 case vignettes this yielded a total of 4,095 triage evaluations. The reported findings suggest that most symptom checkers’ triage performance is not better than the average medical layperson, and that the 15 symptom checker apps in general fare worst where laypersons would require greatest support, that is, in determining when self-care is appropriate. We used the publicly available data set of Schmieding et al [36] to benchmark the triage capability of symptom checkers in 2020 (based on data from Hill et al [24,34] and our data) against that of medical laypersons.

| App | App or Website | URL |
| --- | --- | --- |
| Ada | App | ‎https://apps.apple.com/us/app/ada-your-health-companion/id1099986434 |
| Ask NHS | App | https://apps.apple.com/us/app/ask-nhs-virtual-assistant/id1222121280 |
| Babylon | App | https://apps.apple.com/us/app/ask-nhs-virtual-assistant/id1222121280 |
| Caidr | App | https://apps.apple.com/us/app/ask-nhs-virtual-assistant/id1222121280 |
| Drugs.com | Website | https://www.drugs.com/symptom-checker/ |
| EarlyDoc | Website | https://www.earlydoc.com/en |
| Everyday Health | Website | https://www.everydayhealth.com/symptom-checker/ |
| Family Doctor | Website | https://familydoctor.org/your-health-resources/health-tools/symptom-checker/ |
| Healthdirect | Website | https://www.healthdirect.gov.au/symptom-checker/ |
| Healthily | App | https://apps.apple.com/GB/app/id1491316446/id1491316446?mt=8 |
| HealthTap | App | https://apps.apple.com/GB/app/id1491316446/id1491316446?mt=8 |
| Healthwise | Website | https://myhealth.alberta.ca/Health/pages/symptom-checker.aspx |
| Healthy Children (KidsDoc Symptom Checker) | Website | www.healthychildren.org/English/tips-tools/symptom-checker |
| Isabel | Website | https://symptomchecker.isabelhealthcare.com/ |
| K Health | App | https://apps.apple.com/us/app/k-health-telehealth/id1180400838 |
| Mayo Clinic | Website | https://www.mayoclinic.org/symptom-checker/select-symptom/itt-20009075 |
| NHS Symptom Checker | Website | https://www.nhsdirect.wales.nhs.uk/SelfAssessments/ |
| Quro | Website | https://www.quro.ai/ |
| Symcat | Website | http://www.symcat.com/ |
| Symptify | Website | https://symptify.com/ |
| Symptomate | App | https://apps.apple.com/us/app/symptomate/id837725433 |
| WebMD | Website | <https://apps.apple.com/us/app/webmd-symptoms-doctors-rx/id295076329> |

Supplementary Table 1. Symptom checkers included in our study sample of symptom checker apps capable of triage (n=22).

| App | “Self-care" level avail-able^1^ | Triage performance | | | | | | |
| --- | --- | --- | --- | --- | --- | --- | --- | --- |
|  |  | 3-tiered triage decision^2^ | Binary triage decision | | | | | |
|  |  |  | Whether emergency care is required ^3^ | | | Whether medical care is required ^4^ | | |
|  |  | Acc^5^ | Acc | SN^6^ | SP^7^ | Acc | SN | SP |
| Ada | Yes | 64% (29/45) | 89% (40/45) | 87% (13/15) | 90% (27/30) | 76% (34/45) | 100% (30/30) | 27% (4/15) |
| Ask NHS | Yes | 61% (20/33) | 76% (25/33) | 33% (4/12) | 100% (21/21) | 82% (27/33) | 83% (19/23) | 80% (8/10) |
| Babylon | Yes | 66.6% (28/42) | 88% (37/42) | 71% (10/14) | 96% (27/28) | 79% (33/42) | 100% (29/29) | 31% (4/13) |
| Caidr | Yes | 50% (13/26) | 81% (21/26) | 29% (2/7) | 100% (19/19) | 69% (18/26) | 93% (14/15) | 36% (4/11) |
| Drugs.com | Yes | 75% (30/40) | 80% (32/30) | 50% (7/14) | 96% (25/26) | 92% (37/40) | 100% (28/28) | 75% (9/12) |
| EarlyDoc | Yes | 50% (11/22) | 68% (15/22) | 29% (2/7) | 87% (13/15) | 82% (18/22) | 93% (14/15) | 57% (4/7) |
| Everyday Health | No | 48% (15/31) | 77% (24/31) | 42% (5/12) | 100% (19/19) | 71% (22/31) | 100% (22/22) | 0% (0/9) |
| Familydoctor | Yes | 56% (23/41) | 83% (34/41) | 54% (7/13) | 96% (27/28) | 68% (28/41) | 70% (19/27) | 64% (9/14) |
| Healthdirect | Yes | 47% (19/40) | 75% (30/40) | 43% (6/14) | 92% (24/26) | 67% (27/40) | 81% (22/27) | 38% (5/13) |
| Healthily | Yes | 70% (21/30) | 80% (24/30) | 91% (10/11) | 74% (14/19) | 83% (25/30) | 91% (20/22) | 62% (5/8) |
| HealthTap | Yes | 48% (20/42) | 74% (31/42) | 33% (5/15) | 96% (26/27) | 71% (30/42) | 100% (29/29) | 8% (1/13) |
| Healthwise | Yes | 57% (24/42) | 88% (37/42) | 79% (11/14) | 93% (26/28) | 67% (28/42) | 86% (24/28) | 29% (4/14) |
| Healthy Children | Yes | 86% (12/14) | 86% (12/14) | 100% (3/3) | 82% (9/11) | 100% (14/14) | 100% (8/8) | 100% (6/6) |
| Isabel | No | 56% (25/45) | 89% (40/45) | 80% (12/15) | 93% (28/30) | 67% (30/45) | 100% (30/30) | 0% (0/15) |
| K Health | No | 26% (8/31) | 87% (27/31) | 67% (8/12) | 100% (19/19) | 71% (22/31) | 100% (22/22) | 0% (0/9) |
| Mayo | Yes | 63% (24/38) | 89% (34/38) | 77% (10/13) | 96% (24/25) | 74% (28/38) | 70% (19/27) | 82% (9/11) |
| NHS | Yes | 62% (18/29) | 72% (21/29) | 40% (4/10) | 89% (17/19) | 90% (26/29) | 100% (19/19) | 70% (7/10) |
| Quro | Yes | 44% (19/43) | 74% (32/43) | 21% (3/14) | 100% (29/29) | 70% (30/43) | 93% (27/29) | 21% (3/14) |
| Symcat | No | 43% (19/44) | 70% (31/44) | 47% (7/15) | 83% (24/29) | 68% (30/44) | 100% (30/30) | 0% (0/14) |
| Symptify | Yes | 60% (25/42) | 81% (34/42) | 86% (12/14) | 79% (22/28) | 74% (31/42) | 97% (28/29) | 23% (3/13) |
| Symptomate | Yes | 48% (15/31) | 74% (23/31) | 58% (7/12) | 84% (16/19) | 74% (23/31) | 91% (20/22) | 33% (3/9) |
| WebMD | No | 13% (6/45) | 76% (34/45) | 40% (6/15) | 93% (28/30) | 67% (30/45) | 100% (30/30) | 0% (0/15) |

Supplementary Table 2: Triage performance of 22 symptom checker apps in 2020 tested on 45 case vignettes as compiled by Semigran et al [23]. Symptom checker apps are ordered alphabetically. ^1^ “’Self-care’ level available” denotes whether a symptom checker ever provided advice, that self-care was appropriate. ^2^ The case vignettes gold standard triage solution is either “emergency care required”, “non-emergency care required” or “self-care appropriate” as defined by Semigran et al [23]. ^3^ Symptom checkers’ triage recommendations were counted as correct when they correctly differentiated between cases requiring emergency care and those not requiring emergency care (non-emergency care and self-care). ^4^ Symptom checkers’ triage recommendations were counted as correct when they correctly differentiated between cases requiring medical care (emergency care and non-emergency care) and those not requiring medical care at all (self-care). ^5^ Accuracy. ^6^ Sensitivity. ^7^ Specificity.

| App | Diagnostic accuracy | |
| --- | --- | --- |
|  | M1 | M10 |
| Ada | 53% (24/45) | 71% (32/45) |
| Babylon | 37% (9/24) | 54% (13/24) |
| Everyday Health | 68% (19/28) | 79% (22/28) |
| Familydoctor | 50% (19/38) | 53% (20/38) |
| Healthily | 52% (15/29) | 52% (15/29) |
| HealthTap | 37% (15/40) | 67% (27/40) |
| Isabel | 27% (12/45) | 76% (34/45) |
| K Health | 52% (16/31) | 77% (24/31) |
| Mayo | 26% (10/38) | 74% (28/38) |
| Quro | 33% (14/43) | 65% (28/43) |
| Symcat | 47% (21/45) | 78% (35/45) |
| Symptify | 43% (18/42) | 59% (25/42) |
| Symptomate | 81% (22/27) | 85% (23/27) |
| WebMD | 44% (20/45) | 71% (32/45) |

Supplementary Table 3: Diagnostic performance of 14 symptom checker apps in 2020 tested on 45 case vignettes as compiled by Semigran et al [23]. M1 and M10 refers to the proportion of case evaluations where the symptom checker app listed the gold standard diagnosis (or synonym) as first diagnostic suggestion (M1) or within the first ten diagnostic suggestions (M10), respectively.

| Triage recommendation provided by the symptom checker app | | Gold standard solution of the triage level for the case vignette | | |
| --- | --- | --- | --- | --- |
|  |  | Emergency | Non-  emergency | Self-care |
|  | Emergency care | 80.3%  (147/183) | 37.7%  (66/175) | 24.7%  (43/174) |
|  | Non-emergency | 16.9%  (31/183) | 54.8%  (96/175) | 41.9%  (73/174) |
|  | Self-care | 2.7%  (5/183) | 7.4%  (13/175) | 33.3%  (58/174) |

Supplementary Table 4. Confusion matrix of triage advice of all 15 symptom checker apps providing triage advice assessed by Semigran et al [23].

| Triage recommendation provided by the symptom checker app | | Gold standard solution of the triage level for the case vignette | | |
| --- | --- | --- | --- | --- |
|  |  | Emergency | Non-  emergency | Self-care |
|  | Emergency care | 56.8%  (154/271) | 12.2%  (33/270) | 3.1%  (8/255) |
|  | Non-emergency | 36.1%  (98/271) | 67.4%  (182/270) | 51.3%  (135/255) |
|  | Self-care | 2.2%  (6/271) | 11.8%  (32/270) | 34.5%  (88/255) |
|  | Not emergency care ^1^ | 4.7% (12/271) | 8.5% (23/270) | 9.4% (24/255) |

Supplementary Table 5. Confusion matrix of triage advice of all 22 symptom checker apps assessed in 2020 on the same 45 case vignettes as used by Semigran et al [23]. ^1^ Two apps (*K Health*, *WebMD*) only provided triage advice when they deemed “emergency care” necessary. Their omission of a triage recommendation was thus rated as an appraisal that no emergency care was required.

# Rules on entering the case vignettes into symptom checker apps and rating the symptom checker’s output

1. Chief complaints

Symptom checkers (SC) asking for a chief complaint were provided the chief complaint either assigned by Hill et al [24,34] or by two physicians among the authors of our study (KS, MLS). SCs not asking for a chief complaint were provided the chief complaint as first symptom.

For case evaluations where a chief complaint could not be entered into a SC, but other symptoms from the case vignette could be entered, this evaluation was still counted, unless the symptom checker explicitly returned that based on the provided information no advice can be given.

1. Order of symptom entry

SCs allowing the user to choose in which order to provide symptoms were provided the chief complaint first, all the remaining symptoms in the order in which they appeared in the vignette.

1. Triage advice without a diagnostic suggestion

Case evaluations yielding only either diagnostic or triage advice were counted and not omitted.

1. Information not provided in the vignette but asked for by the SC

When SC asked for information not provided in the modified vignettes, these prompts were answered negatively. For example, if a SC asked whether a fictitious patient has a specific diagnosis as comorbidity and the case vignette did not provide information on this then we responded to the prompt by denying that the fictitious patient suffers from this comorbidity. Assessing SC offering a more “neutral” response such as “I don’t know” next to the options “Yes” and “No”, we still responded with “No” (indicating what was asked for was not present) and not with “I don’t know” or similar ambiguous responses available.

1. Negative symptoms and findings
   Wherever possible negative symptoms in the case vignette (eg, “no shortness of breath”, “no fever”) were provided to the symptom checker apps.
2. Triage recommendations linked to diagnostic suggestions

When SC linked their triage advice to diagnostic suggestions the triage level of the first provided diagnostic suggestion was counted, except if the SC also provided a “summary triage advice”. If the SC required to click on a diagnosis and read through a text hinting at the diagnosis’ urgency without providing immediate advice to the user on whether and where to seek care, this triage advice was not counted.

1. Conditional triage advice

Apps providing conditional triage advice, for example by recommending to seek care if a certain symptom is present, then this was counted as triage advice when the information required to check whether the condition was fulfilled was provided in the case vignette.

# Classification of Advice of triage advice provided by symptom checkers

By and large we followed Semigran et al [23] definitions of the three triage levels “emergency care required”, “non-emergency care required” and “self-care appropriate”. Some symptom checkers provided triage advice that did not self-evidently fit into one of the three definitions by Semigran et al [23]. Thus we had to make minor additions to their definitions:

- Urgency appraisals including the words “dangerous” or “danger” were counted as advice to seek emergency care.
- Advice to “call a hotline” or cautious phrasing such as “you might require advice by a GP” were counted as “non-emergency care” required.
- We counted advice as “self-care sufficient” when the SC used wording along the lines of “You can try self-care”, “You do not need any medical help” and “These symptoms can be treated at home”.
- If the SCs phrased no triage recommendations fitting the above criteria but provided color codes instead, red was rated as requiring emergency care, yellow as requiring non-emergency care and green as self-care being appropriate.

| **Number** | **Gold Standard Urgency Level^1^** | **Gold Standard Diagnosis^2^** | **Original Text of Case Vignette^3^** | **Semigran et al Version^3^** | **Hill et al Version^4^** | **Chief Complaint^4^** | **Supplemented Information and Attributes of the Chief Complaint^5^** |
| --- | --- | --- | --- | --- | --- | --- | --- |
| 1 | Em | Acute liver failure (acetaminophen poisoning acceptable) | A 48-year-old woman with a history of migraine headaches presents to the emergency room with altered mental status over the last several hours. She was found by her husband, earlier in the day, to be acutely disoriented and increasingly somnolent. On physical examination, she has scleral icterus, mild right upper quadrant tenderness, and asterixis. Preliminary laboratory studies are notable for a serum ALT of 6498 units/L, total bilirubin of 5.6 mg/dL, and INR of 6.8. Her husband reports that she has consistently been taking pain medications and started taking additional 500 mg acetaminophen pills several days ago for lower back pain. Further history reveals a medication list with multiple acetaminophen-containing preparations. | 48 y/o f, confusion, disorientation, increasingly drowsy, mild right upper quadrant tenderness, chronic tylenol/acetaminophen - recently took more | 48-year-old female with history of migraines. Confused. Disoriented. Increasingly sleepy. Mild right upper abdomen tenderness. Has been on various pain medications and has been taking more paracetamol over the last few days because of lower back pain. Chief complaint = confusion | Confusion (Alternatively Abdominal pain) | Specifications on the Chief Complaint: Onset: acute, several hours ago; Trend: Worsening, continuous Severity: severe  PMH / Medication: history of migraine, has taken acetaminophen (=tylenol, = ibuprofen)  Added symptoms (from original text): - yellowed eyes (jaundice) - "flapping tremor" (a tremor of the hand when the wrist is extended, sometimes said to resemble a bird flapping its wings) |
| 2 | Em | Appendicitis | A 12-year-old girl presents with sudden-onset severe generalised abdominal pain associated with nausea, vomiting, and diarrhoea. On exam she appears ill and has a temperature of 104°F (40°C). Her abdomen is tense with generalised tenderness and guarding. No bowel sounds are present. | 12 y/o f, sudden onset severe abdominal pain, nausea, vomiting, diarrhea, T=104 | 12-year-old female. Sudden, severe abdominal pain. Feels sick (nausea). Had vomiting and diarrhoea. Temperature 40°C. Chief complaint = abdominal pain | Abdominal Pain | Specifications on the Chief Complaint: Onset: acute, several hours ago; Trend: Worsening, continuous Quality: changed from dull to localized and sharp; Severity: severe  PMH / Medication: None |
| 3 | Em | Asthma (or acute asthma, status asthmaticus, exacerbation of asthma) | A 27-year-old woman with a history of moderate persistent asthma presents to the emergency room with progressive worsening of shortness of breath, wheezing, and cough over 3 days. She reports prior exposure to a person who had a runny nose and a hacking cough. She did not receive significant relief from her rescue inhaler with worsening symptoms, despite increased use. She has been compliant with her maintenance asthma regimen, which consists of an inhaled corticosteroid and a leukotriene receptor antagonist for maintenance therapy and albuterol as rescue therapy. Her cough is disrupting her sleep pattern and as a consequence she is experiencing daytime somnolence, which is affecting her job performance. | 27 y/o f, Hx of asthma, mild shortness of breath, wheezing, 3 days cough, symptoms not responsive to inhalers, recent cold | 27-year-old female. History of asthma. Progressively worsening shortness of breath, wheezing and coughing over 3 days. Not responding to inhalers. Cough disturbing her sleep and is sleepy in the daytime. Chief complaint = shortness of breath/breathing problem | Shortness of Breath / Breathing problems | Specifications on the Chief Complaint: Onset: three days ago Trend: Worsening, continuous Severity: Medium  PMH / Medication: Asthma, has used asthma inhaler  (If asked: bluish lips (cyanosis) |
| 4 | Em | COPD exacerbation (severe) (diagnosis may include acute bronchitis) | A 67-year-old woman with a history of COPD presents with 3 days of worsening dyspnoea and increased frequency of coughing. Her cough is now productive of green, purulent sputum. The patient has a 100-pack-year history of smoking. She has had intermittent, low-grade fever of 100°F (37.7°C) for the past 3 days and her appetite is poor. She has required increased use of rescue bronchodilator therapy in addition to her maintenance medications to control symptoms. | 67 y/o f, Hx of COPD, 3 days worsening shortness of breath, increase coughing, green sputum, low grade fever, increase use of rescue bronchodilator therapy | 67-year-old female. History of chronic obstructive airways disease (COPD). 3 days of worsening shortness of breath and cough with green phlegm. Mild fever. (37.7°C) Poor appetite. Increased use of bronchodilator/salbutamol in addition to normal treatment. Smoker (if asked). Chief complaint = shortness of breath/breathing problem | Shortness of breath / breathing problem (Alternatively Cough) | Specifications on the Chief Complaint: Onset: subacute, within a day, lasting 3d now Trend: worsening, continuous Severity: medium  PMH / Medication: Known COPD, bronchodilator therapy |
| 5 | Em | Deep vein thrombosis | A 65-year-old woman presents with unilateral leg pain and swelling of 5 days' duration. There is a history of  hypertension, mild CHF, and recent hospitalization for pneumonia. She had been recuperating at home but on  beginning to mobilize and walk, the right leg became painful, tender, and swollen. On examination, the right calf is 4  cm greater in circumference than the left when measured 10 cm below the tibial tuberosity. Superficial veins in the  leg are more dilated on the right foot and the right leg is slightly redder than the left. There is some tenderness on  palpation in the popliteal fossa behind the knee. | 65 y/o f, 5 days swelling, pain in one leg, recent hospitalization, leg painful, tender, swollen, red | 65-year-old female. History of high blood pressure and mild congestive heart failure. 5 days of swelling and pain in 1 leg. Recent hospitalisation. Leg is painful, sore, red, swollen. Chief complaint = Leg pain | Leg pain (Alternatively swollen leg) | Specifications on the Chief Complaint: Onset: 5 days ago, insidious; Trend: worsening, continuous Quality: dull Severity: medium  PMH / Medication: Hypertension, mild Congestive Heart failure, recent hospitalisation for pneumonia |
| 6 | Em | Myocardial infarction (heart attack) | Mr. Y is a 64-year-old Chinese male who presents with chest pain for 24 hours. One day before presentation, the patient began to experience 8/10, non-radiating substernal chest pressure associated with diaphoresis and shortness of breath. The pain initially improved with Tylenol, however over the following 24 hours, his symptoms worsened. The patient went to his primary physician, where an EKG was performed which showed ST elevation in leads V2–V6. | 64 y/o m, 1 day chest pain (8/10), non-radiating substernal chest pressure, sweating, shortness of breath, (chest tightness ) | 64-year-old male. 1 day of chest pain (8/10 pain). Pain does not move elsewhere. Sweating. Breathless. Feels tightness in mid chest (include if asked, as this was an added symptom in Semigran article). Chief complaint = chest pain | Chest pain (Alternatively shortness of breath) | Specifications on the Chief Complaint: Onset: 24h ago, suddenly Trend: Worsening, continuously; pain worsened by phyical activity, pain not worsened by applying touch (palpation)  Quality: dull/pressure/tightness Severity: Severe  PMH / Medication: None |
| 7 | Em | Haemolytic uremic syndrome1 | A 4-year-old boy presents with a 7-day history of abdominal pain and watery diarrhoea that became bloody after the first day. Three days before the onset of symptoms, he had visited the county fair with his family and had eaten a hamburger. Physical examination reveals a mild anaemia. | 4 y/o m, 7 day Hx of abdominal pain, bloody diarrhea, ate hamburger at fair 3 days ago | 4-year-old male. 7 days of stomach pain. Blood in diarrhoea. (Only if asked) - Ate a hamburger at a fair 3 days before stomach-ache began. Chief complaint = diarrhoea | Diarrhoea (Alternatively abdominal pain) | Specifications on the Chief Complaint: Onset: 7days ago, suddenly Trend: constant Quality: Pain dull, diarrhoea bloody Severity: Pain severe; diarrhoea severe, leading to dehydration  PMH / Medication: None |
| 8 | Em | Ureteric stones (kidney stones) | A 45-year-old white man presents to the emergency department with a 1-hour history of sudden onset of left-sided flank pain radiating down toward his groin. The patient is writhing in pain, which is unrelieved by position. He also complains of nausea and vomiting. | 45 y/o m, 1 hour severe leftsided flank pain radiating into groin, nausea, vomiting, pain unrelieved by position | 45-year-old male. 1 hour of severe left sided lower back pain radiating to groin. Feels sick. Vomiting. Pain unrelieved by position. Chief complaint = Back pain | Back pain | Specifications on the Chief Complaint: Onset: Sudden, 1h ago Trend: Constant Quality: Sharp, colicky, radiating to groin Severity: Severe  PMH / Medication: None |
| 9 | Em | Malaria | A 28-year-old man presents to his physician with a 5-day history of fever, chills, and rigors, not improving with acetaminophen (paracetamol), along with diarrhoea. He had been traveling in Central America for 3 months, returning 8 weeks ago. He had been bitten by mosquitoes on multiple occasions, and although he initially took malaria prophylaxis, he discontinued it due to mild nausea. He does not know the specifics of his prophylactic therapy. On examination he has a temperature of 100.4°F (38°C) and is mildly tachycardic with a BP of 126/82 mmHg. The remainder of the examination is normal. | 28 y/o m, 5 day Hx of fever, chills, rigors, diarrhea, recent travel abroad to area with malaria, bitten by mosquitoes, did not take malaria prophylaxis consistently | 28-year-old male. 5 days of chills, shivering, diarrhoea. (Only if asked by symptom checker - Recently been overseas to New Guinea. Was bitten by mosquitos. Didn’t regularly take anti-malaria medication). Chief complaint = fever | Fever | Specifications on the Chief Complaint: Onset: Subacute, 5days ago Trend: intermittent /undulating Severity: 38°C  PMH / Medication: None |
| 10 | Em | Meningitis | An 18-year-old male student presents with severe headache and fever that he has had for 3 days. Examination reveals fever, photophobia, and neck stiffness. | 18 y/o m, 3 days severe headache, fever, photophobia, neck stiffness | 18-year-old male. 3 days of severe headache. Fever, sore neck and light sensitivity. Chief complaint = headache | Headache | Specifications on the Chief Complaint: Onset: Acute/Sudden, 3d ago Trend: Worsening, continuous Quality: Dull, whole head affected (holocephalic) Severity: Severe   PMH / Medication: None |
| 11 | Em | Pneumonia | A 65-year-old man with hypertension and degenerative joint disease presents to the emergency department with a three-day history of a productive cough and fever. He has a temperature of 38.3°C (101°F), a blood pressure of 144/92 mm Hg, a respiratory rate of 22 breaths per minute, a heart rate of 90 beats per minute, and oxygen saturation of 92 percent while breathing room air. Physical examination reveals only crackles and egophony in the right lower lung field. The white-cell count is 14,000 per cubic millimeter, and the results of routine chemical tests are normal. A chest radiograph shows an infiltrate in the right lower lobe. | 65 y/o m, Hx of hypertension and degenerative joint disease, 3 day Hx of productive cough and fever (101) | 65-year-old male. History of high blood pressure and degenerative joint disease. Had 3 days of coughing up phlegm with a fever. Temperature 38.3°C. Chief complaint = cough | Cough (Alternatively Fever) | Specifications on the Chief Complaint: Onset: Acute, within hours, lasting 3d Trend: Worsening Quality: Productive, yellow sputum   PMH / Medication: Chronic hypertension, degenerative joint disease (e.g. rheumatoid arthritis) |
| 12 | Em | Pulmonary embolism | A 65-year-old man presents to the emergency department with acute onset of short of breath of 30 minutes’ duration. Initially, he felt faint but did not lose consciousness. He is complaining of left-sided chest pain that worsens on deep inspiration. He has no history of cardiopulmonary disease. A week ago, he underwent a total left hip replacement and, following discharge, was on bed rest for 3 days due to poorly controlled pain. He subsequently noticed swelling in his left calf, which is tender on examination. His current vital signs reveal a fever of 100.4°F (38.0°C), heart rate 112 bpm, BP 95/65, and an O2 saturation on room air of 91%. | 65 y/o m, shortness of breath for 30 min, chest pain that worsens with inspiration, recent surgery, recent bed rest, swelling in left calf, which is tender, fever | 65-year-old male. Breathless for last 30 minutes. Chest pain on left side which is worse when breathing in. Had recent surgery, with bedrest. Has a swollen left calf which is painful. Has a fever. No history of heart disease.  Chief complaint = shortness of breath/breathing problem | shortness of breath/breathing problem | Specifications on the Chief Complaint: Onset: 30min ago, sudden Trend: worsening, worsening on inspiration Quality: sharp (pleuritic) Severity: severe  PMH / Medication: None |
| 13 | Em | Rocky Mountain Spotted Fever | An 8-year-old boy in Oklahoma is brought to the emergency department over the fourth of July weekend because of fever, chills, malaise, athralgias, and a headache. Physical examination reveals a maculopapular rash that is most prominent on his wrists and ankles. | 8 y/o m, Fever, chills, joint pain, headache, rash wrists/ankles | NA | Fever (Alternatively Headache, Joint pain) | Specifications on the Chief Complaint: Onset: Sudden (within an hour), 2days ago Trend: constant Severity: Peaking at 39°C   PMH / Medication: None  Geographical Location: USA |
| 14 | Em | Stroke | A 70-year-old man with a history of chronic hypertension and atrial fibrillation is witnessed by a family member to have nausea, vomiting, and right-sided weakness, as well as difficulty speaking and comprehending language. The symptoms started with only mild slurred speech before progressing over several minutes to severe aphasia and right arm paralysis. The patient is taking warfarin. | 70 y/o m, nausea, vomiting, right-sided weakness, rt arm paralysis, difficulty speaking and comprehension | 70-year-old male. History of high blood pressure and atrial fibrillation. Feels sick. Vomiting. Weak down right side. Right arm paralysed. Has trouble speaking and is confused. Takes warfarin. Chief complaint = weak right arm | Weak right arm (Alternatively Confusion, Problem speaking) | Specifications on the Chief Complaint: Onset: Sudden, several minutes Trend: Worsening Severity: severe  PMH / Medication: Chronic hypertension, atrial fibrillation, Warfarin  Add: apart from slurred speech: aymmetric facial expression; can't smile symmetrically |
| 15 | Em | Tetanus | A 63-year-old man sustained a cut on his hand while gardening. His immunisation history is significant for not having received a complete tetanus immunisation schedule. He presents with signs of generalised tetanus with trismus (“lock jaw”), which results in a grimace described as “risus sardonicus” (sardonic smile). Intermittent tonic contraction of his skeletal muscles causes intensely painful spasms, which last for minutes, during which he retains consciousness. The spasms are triggered by external (noise, light, drafts, physical contact) or internal stimuli, and as a result he is at the risk of sustaining fractures or developing rhabdomyolysis. The tetanic spasms also produce opisthotonos, board-like abdominal wall rigidity, dysphagia, and apnoeic periods due to contraction of the thoracic muscles and/or glottal or pharyngeal muscles. During a generalised spasm the patient arches his back, extends his legs, flexes his arms in abduction, and clenches his fists. Apnoea results during some of the spasms. Autonomic overactivity initially manifests as irritability, restlessness, sweating, and tachycardia. Several days later this may present as hyperpyrexia, cardiac arrhythmias, labile hypertension, or hypotension. | 65 y/o m, cannot open mouth, contraction of muscles causing painful spasms for minutes, sweating, tachycardia, cut hand while gardening, did not get tetanus shot | 65-year-old male. Cannot open mouth. Muscles tightened, causing painful spasms for minutes at a time. Sweating. Fast heart rate. Has cut his hand while gardening and did not get a tetanus injection. Chief complaint = muscle spasms | Muscle spasms Alternatively Lockjaw / Cannot open mouth, Muscle pain, Sweating | Specifications on the Chief Complaint: Onset: Subacute, over eight days Trend: Worsening Severity: severe  PMH / Medication: None, no tetanus vaccination |
| 16 | NE | Acute otitis media | An 18-month-old toddler presents with 1 week of rhinorrhea, cough, and congestion. Her parents report she is irritable, sleeping restlessly, and not eating well. Overnight she developed a fever. She attends day care and both parents smoke. On examination signs are found consistent with a viral respiratory infection including rhinorrhea and congestion. The toddler appears irritable and apprehensive and has a fever. Otoscopy reveals a bulging, erythematous tympanic membrane and absent landmarks. | 18 mo f, 1 week rhinorrhea, cough, congestion, irritable, lack of appetite, fever, in daycare | 18-month-old female. 1 week of runny nose, cough, feels chesty, irritable. No appetite. Fever. Goes to day care. Chief complaint = runny nose | Runny Nose (Alternatively Cough, Fever) | Specifications on the Chief Complaint: Onset: Subacute, within days, lasting 8d Trend: Worsening  PMH / Medication: None |
| 17 | NE | Acute pharyngitis | A 7-year-old girl presents with abrupt onset of fever, nausea, vomiting, and sore throat. The child denies cough, rhinorrhea, or nasal congestion. On physical exam, oral temperature is 101°F (38.5°C) and there is an exudative pharyngitis, with enlarged cervical lymph nodes. A rapid antigen test is positive for group A Streptococcus (GAS). | 7 y/o f, fever (101), nausea, vomiting, sore throat, swollen lymph nodes, tonsilar exudate; no cough, rhinorrhea, or nasal congestion | 7-year-old female. Fever (38.5°C). Feels sick. Vomiting. Sore throat, swollen neck glands. Tonsils have visible pus. No cough, no runny nose nor blocked nose. Chief complaint = fever | Fever (Alternatively Sore throat) | Specifications on the Chief Complaint: Onset: Abrupt / Acute Trend: Worsening Severity: (101°F) 38.5°C  PMH / Medication: None |
| 18 | NE | Acute pharyngitis | Mr. A is a 24 year-old man who presents to your office for complaints of sore throat, fever, and headache. His symptoms started 2 days ago with acute onset of sore throat and fever to 102.2. He has had no cough. His physical examination is normal, except for the presence of tonsillar exudates and some tender anterior cervical lymphadenopathy. He is otherwise in good health, and is on no medications except for ibuprofen for fever. He has no drug allergies. (, Centor score = 4 – treat, or test and treat) | 24 y/o m, sore throat, fever (102.2), headache, no cough,tonsilar exudates | NA | Fever (Alternatively Sore throat) | Specifications on the Chief Complaint: Onset: Abrupt, two days Trend: Constant Severity: 102.2°F  PMH / Medication: None |
| 19 | NE | Acute sinusitis | Mrs. S is a 35 year-old woman who presents with 15 days of nasal congestion. She has had facial pain and green nasal discharge for the last 12 days. She has had no fever. On physical examination, she has no fever and the only abnormal finding is maxillary tenderness on palpation. She is otherwise healthy, except for mild obesity. She is on no medications, except for an over-the-counter decongestant. She has no drug allergies | 35 y/o f, sx for 15 days, nasal congestion, facial pain, green nasal discharge, no fever | 35-year-old female. Unwell for 15 days. Has blocked nose with green mucous and facial pain for last 12 days. No fever. Chief complaint = nasal congestion | Nasal congestion (Alternatively Facial Pain) | Onset: Acute, whithin hours, lasting 15days, Trend: constant, continuous Severity: worsening  PMH / Medication: Mild obesity, OTC decongestant |
| 20 | NE | Back pain | Consider a 35-year-old man who developed low back pain after shoveling snow 3 weeks ago. He presents to the office for an evaluation. On examination there is a new left foot drop. In study 82% physicians recommend MRI (sciatica/sprain) | 35 y/o m, back pain following shoveling, left foot drop, symptoms 3 weeks of duration (loss of sensation in foot) | 35-year-old male. Back pain following shovelling 3 weeks ago. (Only if asked - Left foot has gone numb and is weak. This was an added symptom in Semigran article). Chief complaint = back pain | Back pain | Specifications on the Chief Complaint: Onset: Acute, whithin minutes, lasting 3w, Trend: varying, exacerbated by lifting weights and prolonged sitting/standing or coughing Quality: radiating into leg, "aching", burning Severity: medium  PMH / Medication: None |
| 21 | NE | Cellulitis | A 45-year-old man presents with acute onset of pain and redness of the skin of his lower extremity. Low-grade fever is present and the pretibial area is erythematous, edematous, and tender. | 45 y/o m, pain and redness of skin, low grade fever, redness, edema, and tenderness lower leg | 45-year-old male. Pain, swollen, sore and redness of skin in lower leg. Mild fever. Chief complaint = leg pain | Leg pain | Specifications on the Chief Complaint: Onset: acute, within a few hours Trend: worsening, continuous Quality: throbbing, tender (worsening with touch and movement), sharp, one-sided! Severity: medium  PMH / Medication: None |
| 22 | NE | COPD flare | A 56-year-old woman with a history of smoking presents to her primary care physician with shortness of breath and cough for several days. Her symptoms began 3 days ago with rhinorrhea. She reports a chronic morning cough productive of white sputum, which has increased over the past 2 days. She has had similar episodes each winter for the past 4 years. She has smoked 1 to 2 packs of cigarettes per day for 40 years and continues to smoke. She denies hemoptysis, chills, or weight loss and has not received any relief from over-the-counter cough preparations. | 56 y/o f, Hx of smoking, shortness of breath and cough for several days, rhinorrhea 3 days ago, white sputum, no chills | NA | shortness of breath/breathing problem (Alternatively Cough) | Specifications on the Chief Complaint: Onset: Subacute, within a day, lasting 3 days Trend: Constant Severity: Mild  PMH / Medication: None  Added explicitly: No Fever |
| 23 | NE | Influenza | A 30-year-old woman presents in January with 2-day history of fever, cough, headache, and generalized weakness. She was in her usual state of health before an abrupt onset of these symptoms. A few viral illnesses have affected her during the current winter, but not to this severity. She reports sick contacts at work and did not receive the seasonal influenza vaccine this season. | 30 y/o f, 2 day fever, cough, headache, weakness, did not get flu shot | NA | Fever (Alternatively Cough) | Specifications on the Chief Complaint: Onset: Acute, within an hour Trend: Constant Severity: 38.5°C (101.3°F)  PMH / Medication: None |
| 24 | NE | Mononucleosis | A 16-year-old female high school student presents with complaints of fever, sore throat, and fatigue. She started feeling sick 1 week ago. Her symptoms are gradually getting worse, and she has difficulty swallowing. She has had a fever every day, and she could hardly get out of bed this morning. She does not remember being exposed to anybody with a similar illness recently. On physical examination she is febrile and looks sick. Enlarged cervical lymph nodes, exudative pharyngitis with soft palate petechiae and faint erythematous macular rash on the trunk and arms are found. | 16 y/o f, 1 week Hx of fever, sore throat, fatigue, difficulty swallowing, fever, enlarged lymph nodes, exudates, macular rash on trunk/arms | 16-year-old female. 1-week history of fever, sore throat, fatigue, difficulty swallowing, unable to get out of bed. Chief complaint = fever | Fever (Alternatively Sore throat) | Specifications on the Chief Complaint: Onset: acute, within hours Trend: Worsening Severity: 38.5°C (101.3°F)  PMH / Medication: None |
| 25 | NE | Peptic ulcer disease | A 40-year-old man presents to his primary care physician with a 2-month history of intermittent upper abdominal pain. He describes the pain as a dull, gnawing ache. The pain sometimes wakes him at night, is relieved by food and drinking milk, and is helped partially by ranitidine. He had a similar but milder episode about 5 years ago, which was treated with omeprazole. Physical examination reveals a fit, apparently healthy man in no distress. The only abnormal finding is mild epigastric tenderness on palpation of the abdomen. | 40 y/o m, 2 month Hx of intermittent upper abdominal pain, dulling and gnawing ache, wakes at night and is relieved by food/drinking milk/ranitidine, prior episode 5 yrs ago | 40-year-old male. 2-month history of intermittent upper abdominal pain. (Dull and gnawing ache). Wakes at night and feels better with food, drink, milk or antacid/Gaviscon. Had a similar experience 5 years ago. Chief complaint = abdominal pain | Abdominal pain | Specifications on the Chief Complaint: Onset: complaints began two months ago, pain episodes begin within less than an hour Trend: intermittent, worsening over time Quality: dull, gnawing ache; relieved by food or beverage intake Severity: medium  PMH / Medication: None |
| 26 | NE | Pneumonia | A 6-year-old boy with a medical history significant for mild persistent asthma is brought to the clinic by his mother with a history of a 5-day cough. His mother reports that the child's fever continues to be elevated despite acetaminophen therapy. He has missed school for the past 3 days and he has a classmate sick with pneumonia. The mother reports that the appetite is good for the child. His cough produced yellowish sputum at home. His vitals at the clinic are: respiratory rate 19 breaths/min, heart rate 80 beats/min, and temperature 101.6°F (38.7°C). He appears in no respiratory distress. His lung examination reveals bilateral rales and occasional wheeze. CXR reveals lobar infiltrates without pleural effusions. | 6 y/o m, Hx of asthma, 5 days cough, fever, appetite good, yellow sputum, t 101.6 | NA | Cough (Alternatively Fever) | Specifications on the Chief Complaint: Onset: subacute within hours, lasting 5 days Trend: constant Quality: yellow sputum Severity: medium  PMH / Medication: Mild persistent asthma, Paracetamol now |
| 27 | NE | Salmonella | A 14-year-old boy presents with nausea, vomiting, and diarrhea. Eighteen hours earlier, he had been at a picnic where he ingested undercooked chicken along with a variety of other foods. He reports moderate-volume, nonbloody stools occurring 6 times a day. He has mild abdominal cramps and a low-grade fever. He is evaluated at an acute care clinic and found to be mildly tachycardic (heart rate 105 bpm) with a normal BP and a low-grade temperature of 100.1°F (37.8°C). His physical exam is unremarkable except for mild diffuse abdominal tenderness and mild increased bowel sounds. He is able to take oral fluids and is instructed on the appropriate oral fluid and electrolyte rehydration. | 14 y/o m, nausea, vomiting, non-bloody diarrhea, mild abdominal cramps (T=100.1), mild abdominal tenderness, diarrhea after attending a picnic and eating undercooked chicken, | NA | Vomiting (Alternatively Diarrhea) | Specifications on the Chief Complaint: Onset: Acute, within hours lasting 18h Trend: worsening Quality: non-bloody, no coffee grounds Severity: medium  PMH / Medication: None |
| 28 | NE | Shingles | A 77-year-old man reports a 5-day history of burning and aching pain on the right side of his chest. This is followed by the development of erythema and a maculopapular rash in this painful area, accompanied by headache and malaise. The rash progressed to develop clusters of clear vesicles for 3 to 5 days, evolving through stages of pustulation, ulceration, and crusting. | 77 y/o m, 5 day burning and aching on right side of chest, erythema, maculopapular rash, headache, malaise, rash progressed to clear vesicles after 3-5 days | 7-year-old male. 5 days of burning and pain on right side of chest. Chest is red with a rash, some spots are clear raised bumps, while some are red and flat. Has a headache and feels tired and unwell. Chief complaint = chest pain | Chest pain (Alternatively Rash) | Specifications on the Chief Complaint: Onset: pain began within a day, 5 days ago. Trend: intermittently worse, constantly present Quality: burning Severity: severe  PMH / Medication: None |
| 29 | NE | Urinary tract infection | A 26-year-old female newly wed presents complaining of painful urination, feeling of urgent need to urinate, and more frequent urination for 2 days. She denies any fever, chills, nausea, vomiting, back pain, vaginal discharge, or vaginal pruritus. | 26 y/o f, painful urination, urgent need to urinate, more frequent urination for 2 days, sexually active; no fever, chills, nausea, vomiting, back pain, vaginal discharge, vaginal pruritus | NA | Painful urination | Specifications on the Chief Complaint: Onset: acute within hours, lasting 2 days Trend: constant Quality: burning, radiating into to center of pelvis, not into the flanks/back Severity: Medium  PMH / Medication: None |
| 30 | NE | Vertigo | A 65-year-old woman presents with a chief complaint of dizziness. She describes it as a sudden and severe spinning sensation precipitated by rolling over in bed onto her right side. Symptoms typically last <30 seconds. They have occurred nightly over the last month and occasionally during the day when she tilts her head back to look upward. She describes no precipitating event prior to onset and no associated hearing loss, tinnitus, or other neurologic symptoms. Otologic and neurologic examinations are normal except for the Dix-Hallpike maneuver, which is negative on the left but strongly positive on the right side. | 5 y/o f, dizziness, sudden onset, recurrent, lasts <30 sec, consistent trigger, no hearing loss, ringing in ears, muscle weakness, loss of sensation | NA | Dizziness | Specifications on the Chief Complaint: Onset: Peracute, within seconds, triggered with movement Trend: Intermittently (<30s), but constant in severity Quality: spinning sensation Severity: Medium  PMH / Medication: None  Added: Explicitly no loss of sensory or motor function! |
| 31 | S-c | Acute bronchitis | A 34-year-old woman with no known underlying lung disease 12-day history of cough. She initially had nasal congestion and a mild sore throat, but now her symptoms are all related to a productive cough without paroxysms. She denies any sick contacts. On physical examination she is not in respiratory distress and is afebrile with normal vital signs. No signs of URI are noted. Scattered wheezes are present diffusely on lung auscultation. | 34 y/o f, 12 day cough, initial nasal congestion and sore throat, cough, no fever | 34-year-old female. 12 days of coughing. Initially had blocked nose and sore throat. Now has cough which brings up phlegm, but no fever. Chief complaint = cough | Cough | Specifications on the Chief Complaint: Onset: subacute, within a few days, lasting 1 2 days Trend: constant Quality: productive, white sputum, non-bloody, non-purulent Severity: mild  PMH / Medication: None |
| 32 | S-c | Acute bronchitis | Mrs. L is a 61 year-old woman who presents with 4 days of a cough productive of yellow sputum. Her symptoms started 4 days ago with rhinorrhea and productive cough. She initially had fevers as high as 101 for 2 days, but those have now resolved. In the office, she has normal vital signs and a normal physical examination. She is otherwise healthy except for high cholesterol for which she is being treated with atorvastatin. She has no drug allergies. | 61 y/o f, 4 day cough, yellow sputum, rhinorrhea, fever (resolved) | NA | Cough (Alternatively Runny nose) | Specifications on the Chief Complaint: Onset: subacute, within a few days, lasting 4 days Trend: getting better Quality: Productive, yellow, non-bloody, non-purulent Severity: mild  PMH / Medication: High cholesterol, atorvastatin |
| 33 | S-c | Acute conjunctivitis | A 14-year-old boy with no significant past medical history presents 3 days after developing a red, irritated right eye that spread to the left eye today. He has watery discharge from both eyes and they are stuck shut in the morning. He reports recent upper respiratory symptoms and that several children at his day camp recently had pink eye. He denies significant pain or light sensitivity and does not wear contact lenses. On examination, his pupils are equal and reactive and he has a right-sided, tender preauricular lymph node. Penlight examination does not reveal any corneal opacity. | 14 y/o m, 3 days red, irritated eye (spread from right to left), discharge, URI symptoms, no pain or light sensitivity | 14-year-old male. 3 days with red irritated eye (spread from right to left eye). Eyes have pus from ducts. Has recently had cold like symptoms. No pain or light sensitivity. Chief complaint = red eye | Red Eye | Specifications on the Chief Complaint: Onset: Acute, within a few hours, present for three days Trend: Progressing to to the other eye, intensity constant Quality: No ciliary flush (red ring around cornea), otherwise distribution even among sclera (white part of eye). Severity: mild  PMH / Medication: None |
| 34 | S-c | Acute pharyngitis | Mr. E is a 26 year-old man who presents to your office for complaints of sore throat, headache, and non-productive cough. His symptoms started 2 days ago with acute onset of sore throat. He has been afebrile. His physical examination is normal, except for some pharyngeal erythema. He is otherwise in good health, and is on no medications except for acetaminophen for his sore throat and fever. He has no drug allergies. | 26 y/o m, 2 day sore throat, headache, cough, no fever | NA | Sore throat (Alternatively cough) | Specifications on the Chief Complaint: Onset: Acute, within days, lasting 2 days Trend: constant Severity: mild  (Cough unproductive)  PMH / Medication: Paracetamol now |
| 35 | S-c | Acute rhinitis | A 22-year-old student presents with a 5-year history of worsening nasal congestion, sneezing, and nasal itching. Symptoms are year-round but worse during the spring season. On further questioning it is revealed that he has significant eye itching, redness, and tearing as well as palate and throat itching during the spring season. He remembers that his mother told him at some point that he used to have eczema in infancy. | 22 y/o m, 5 year Hx of nasal congestion, sneezing, nasal itching worse during spring season, eye itching, redness, tearing, palate and throat itching, Hx of eczema in infancy | 22-year-old male. 5-year history of blocked nose, sneezing, nasal itching which is worse in spring. Has itchy eyes which are red and watery. Throat and palate are also itchy. Has a history of eczema in early childhood. Chief complaint = blocked nose | Nasal congestion (Alternatively Sneezing) | Specifications on the Chief Complaint: Onset: Acute an hour, episodes lasting months-years Trend: intermittent, seasonally worsening, triggered outside Severity: mild   PMH / Medication: Eczema in infancy |
| 36 | S-c | Back pain | A 38-year-old man with no significant history of back pain developed acute LBP when lifting boxes 2 weeks ago. The pain is aching in nature, located in the left lumbar area, and associated with spasms. He describes previous similar episodes several years ago, which resolved without seeing a doctor. He denies any leg pain or weakness. He also denies fevers, chills, weight loss, and recent infections. Over-the-counter ibuprofen has helped somewhat, but he has taken it only twice a day for the past 3 days because he does not want to become dependent on painkillers. On examination, there is decreased lumbar flexion and extension secondary to pain, but a neurologic exam is unremarkable. | 38 y/o m, acute low back pain after lifting, no leg pain or weakness, no fevers, chills, weight loss, or recent infections | 38-year-old male. Sudden lower back pain after lifting. No leg pain or weakness, no fevers or chills, no weight loss or recent infections. Chief complaint = back pain | Back pain | Specifications on the Chief Complaint: Onset: acute, within hours, lasting 2 weeks Trend: overall constant, associated with spasms Quality: aching, associated with spasms, not radiating into the leg Severity: medium  PMH / Medication: None |
| 37 | S-c | Bee sting | A 9-year-old boy is brought to the ER after being stung by a bee at a picnic. He is crying hysterically. After 15 minutes of calming him down, exam reveals a swollen tender upper lip but no tongue swelling, no drooling, no stridor, no rash, and no other complaints. | 9 y/o m, bee sting, swollen and tender upper lip; no tongue swelling, drooling, stridor, rash, or other complaints | 9-year-old male. Stung by a bee, swollen and sore upper lip. No tongue swelling, drooling, noisy breathing, rash or other complaints. Chief complaint = bee sting | Bee sting (Alternatively: Swollen lip) | Specifications on the Chief Complaint: Onset: subacute, within minutes-seconds, lasting about an hour Trend: constant Quality: aching, stabbing; very localised Severity: medium  PMH / Medication: None   Added: explicitly no shortness of breath, wheezing or bluish lips (cyanosis). |
| 38 | S-c | Canker sore | A 17-year-old male student presents with recurrent mouth ulceration since his early schooldays. He has no respiratory, anogenital, gastrointestinal, eye, or skin lesions. His mother had a similar history as a teenager. The social history includes no tobacco use and virtually no alcohol consumption. He has no history of recent drug or medication ingestion. Extraoral exam reveals no significant abnormalities and specifically no pyrexia; no cervical lymph node enlargement; nor cranial nerve, salivary, or temporomandibular joint abnormalities. Oral exam reveals a well-restored dentition and there is no clinical evidence of periodontal-attachment loss or pocketing. He has five 4 mm round ulcers with inflammatory haloes in his buccal mucosae. | 17 y/o m with recurrent mouth ulceration for year, no respiratory, anogenital, gastrointestinal, eye, or skin lesions, mother has similar Hx, no Hx of recent drugs or medication | 17-year-old male. Reoccurring mouth ulcers for a year. No respiratory, anal or genital, gastrointestinal, eye or skin lesions. Mother has similar history. No history of drugs or medications. Chief complaint = mouth ulcers | Mouth ulcers | Onset: since childhood, appear within a day Trend: lasting 7-10d   Sevrity: mild PMH / Medication: None |
| 39 | S-c | Candidal yeast infection | Consider a 40-year-old, monogamous, married woman who calls to report a 2-day history of vaginal itching and thick white discharge. She has no abdominal pain or fever. (in study 50% recommended physician visit) | 40 y/o f, 2 day vaginal itching, thick white discharge, no abdominal pain or fever | NA | Vaginal itching (Alternatively vaginal discharge) | Specifications on the Chief Complaint: Onset: appears within hours, lasting 2 days Trend: constant Severity: mild  PMH / Medication: None |
| 40 | S-c | Constipation | A 5-month-old baby boy presents with difficulty and delay in passing hard stools. His mother reports that he strains for several hours and may even miss a day, before passing stool with screaming and occasional spots of fresh blood on the stool or diaper. He has recently been weaned from breastfeeding to cows' milk formula, which he had been reluctant to drink initially. The child is thriving and now feeding normally. There was no neonatal delay in defecation and no history of excessive vomiting or abdominal distension. | 5 mo m, difficulty/delay in passing hard stools, strains for hours, may miss a day, screams when passes stool and occasional spots of blood, weaned from breastmilk to cows' milk, now feeding normally | NA | Constipation | Specifications on the Chief Complaint: Onset: insidious/chronic onset, lasting days-weeks Trend: constant   PMH / Medication: None |
| 41 | S-c | Eczema | A 12-year-old female presents with dry, itchy skin that involves the flexures in front of her elbows, behind her knees, and in front of her ankles. Her cheeks also have patches of dry, scaly skin. She has symptoms of hay fever and has recently been diagnosed with egg and milk allergy. She has a brother with asthma and an uncle and several cousins who have been diagnosed with eczema. | 12 y/o f, dry, itchy skin in front of elbows, behind knees, in front of ankles, cheeks have patches of dry, scaly skin, symptoms of hay fever, egg and milk allergy, brother has asthma and uncle and cousins have eczema | 12-year-old female. Dry, itchy skin in front of elbows, in front of knees and cheeks have patches of dry, scaly skin. Symptoms of hay fever. Has egg and milk allergy. Brother has asthma and Uncle and cousins have eczema. Chief complaint = rash | Rash | Specifications on the Chief Complaint: Onset: Within days, lasting weeks-months, Trend: Constant, itch is intermittently worse Quality: Itching Severity: Medium  PMH / Medication: None |
| 42 | S-c | Stye | A 30-year-old man presents with a painful, swollen right eye for the past day. He reports minor pain on palpation of the eyelid and denies any history of trauma, crusting, or change in vision. He has no history of allergies or any eye conditions and denies the use of any new soaps, lotions, or creams. On exam, he has localized tenderness to palpation and erythema on the midline of the lower eyelid near the lid margin. The remainder of the physical exam, including the globe, is normal. | 30 y/o m, painful, swollen right eye for past day, no Hx of trauma, crusting, change in vision, allergies, or eye conditions, localized tenderness, erythema (redness) | NA | Painful Eye (Alternatively Red Eye) | Specifications on the Chief Complaint: Onset: Subacute, within a (few) day(s), lasting a day Trend: constant Quality: aching Severity: mild  PMH / Medication: None |
| 43 | S-c | Viral upper respiratory tract infection | Mr. R. 5is a 56 year-old man who presents to you with 6 days of non-productive cough, nasal congestion, and green  nasal discharge. He has had intermittent fevers as high as 100.8. His physical examination is normal except for  rhinorrhea. He is otherwise healthy, except for chronic osteoarthritis of the right knee. He has no drug allergies. | 56 y/o m, 6 day cough, nasal congestion, green nasal discharge, fever (100.8), rhinorrhea | 56-year-old male, 6-day cough, nasal congestion, green nasal discharge. Fever (38.2°C) and runny nose. Chief complaint = cough | Cough (Alternatively nasal congestion) | Onset: Subacute, within a few hours, lasting 6days Trend: constant Quality: non-productive Severity: medium  PMH / Medication: Chronic osteoarthritis of the right knee |
| 44 | S-c | Viral upper respiratory tract infection | A 30-year-old man presents with a 2-day history of runny nose and sore throat. He feels hot and sweaty, has a mild headache, is coughing up clear sputum and complains of muscle aches. He would like antibiotics as he was prescribed them last year when he had a similar condition. On examination, he is afebrile, has a normal pulse, a slightly inflamed pharynx and nontender cervical lymphadenopathy. There is no neck stiffness and his chest is clear. He has tried over-the-counter cough medications, but has not found these helpful. He smokes 10 cigarettes per day. | 30 y/o m, 2 day HX of runny nose, sore throat, hot, sweaty, mild headache, cough with clear sputum, muscle aches, no fever or neck stiffness | NA | Runny and congested nose (Alternatively Cough, Sore throat) | Specifications on the Chief Complaint: Onset: lasting 2 days, developed within a few hours Trend: constant Quality: no pus coating of tonsils (keine Beläge); clear-white mucus Severity: mild  PMH / Medication: Smoker, OTC cough medication |
| 45 | S-c | Vomiting | Elizabeth’s 2-year-old son has a fever and vomited twice. Elizabeth worries about dehydration, so she gives Jack a sippy cup of apple juice. He immediately vomits up the juice. Elizabeth debates what to do next. Should she try to reach Jack’s pediatrician or should she take Jack to the ED? Instead, she calls her triage nurse line. Temperature = 100.5 | 2 y/o m, low grade fever (T = 100.5), vomited twice, vomits up juice | NA | Vomiting | Specifications on the Chief Complaint: Onset: Acute, within a few hours; Trend: Constant   PMH / Medication: None |

Supplementary Table 6. Numbered case vignettes as used by Semigran et al (2015) [23], Hill et al (2020) [24,34] and modified by us with gold standard solutions for diagnosis and urgency level. ^1 “^Em”, “NE” and “S-c” abbreviate the three urgency levels as defined by Semigran et al [23] “emergency care required”, “non-emergency care required” and “self-care appropriate”. We retained the gold standard urgency level as set by Semigran et al [23]. ^2^ We retained the gold standard diagnosis as set by Semigran et al [23], complementing some by additional acceptable diagnoses as suggested by Hill et al [24,34]. ^3^ Complete and abbreviated version of the case vignettes as laid out in the appendix of Semigran et al [23]. See their list of references for the sources of the individual vignettes. Vignettes as modified by Hill et al [24,34] and chief complaints as assigned to the vignettes by Hill et al [24,34]. ^5^ Information supplemented by two physicians (authors KS and MLS), especially attributes of the chief complaints, which we anticipated most symptom checker apps would prompt the mock user to provide.
